# Design and analysis of trials with a partially nested design and a binary outcome measure

**DOI:** 10.1002/sim.6828

**Published:** 2015-12-15

**Authors:** Chris Roberts, Evridiki Batistatou, Stephen A. Roberts

**Affiliations:** ^1^ Centre for Biostatistics, Institute of Population Health, Jean McFarlane BuildingUniversity of ManchesterOxford RoadManchesterM13 9PLU.K.

**Keywords:** partially nested trials, clustering, binary outcomes, sample size, power

## Abstract

Where treatments are administered to groups of patients or delivered by therapists, outcomes for patients in the same group or treated by the same therapist may be more similar, leading to clustering. Trials of such treatments should take account of this effect. Where such a treatment is compared with an un‐clustered treatment, the trial has a partially nested design. This paper compares statistical methods for this design where the outcome is binary.

Investigation of consistency reveals that a random coefficient model with a random effect for group or therapist is not consistent with other methods for a null treatment effect, and so this model is not recommended for this design. Small sample performance of a cluster‐adjusted test of proportions, a summary measures test and logistic generalised estimating equations and random intercept models are investigated through simulation. The expected treatment effect is biased for the logistic models. Empirical test size of two‐sided tests is raised only slightly, but there are substantial biases for one‐sided tests. Three formulae are proposed for calculating sample size and power based on (i) the difference of proportions, (ii) the log‐odds ratio or (iii) the arc‐sine transformation of proportions. Calculated power from these formulae is compared with empirical power from a simulations study.

Logistic models appeared to perform better than those based on proportions with the likelihood ratio test performing best in the range of scenarios considered. For these analyses, the log‐odds ratio method of calculation of power gave an approximate lower limit for empirical power. © 2015 The Authors. Statistics in Medicine published by John Wiley & Sons Ltd.

## 1 Introduction

Methods of statistical analysis used to estimate treatment effects in clinical trials generally assume that subjects are independent. The implications of violations of this assumption in cluster randomised trials are widely recognised with the lack of independence among subjects in the same cluster, reducing precision and causing loss of power. It is now acknowledged that clustering effects should also be considered in the design and analysis of trials of group‐administered treatments and care‐provider activities such as talking or physical therapies 1, 2, 3, 4, 5.

Examples of group‐administered treatments could include classes or group therapy for weight reduction, smoking cessation, alcohol treatment or anger management. While for some such treatments, group administration is simply a matter of efficiency, for example, physical exercise classes involving instruction, in others, synergy between patients can be an active component of the group treatment, examples of which might be self‐help and mutual support groups. Where there is interaction between participants, there are strong theoretical reasons to expect clinical outcomes to be more similar for patients in the same therapy group than for those in different groups. Even with more directive groups, participation and engagement with treatment may depend on others members of the group. Design and analysis of trials of group therapies should therefore presume lack of independence of subjects within therapy groups. Likewise, it is realistic to expect outcomes for patients treated by the same therapist to be more similar, where the outcome of care depends on skill, training, experience or empathy between the patient and the care‐provider. This suggests clustering of patients within care‐provider is also plausible, even where treatments are administered individually.

While there are similarities between statistical methods used where clustering is due to randomisation and where it arises from treatment, there are important differences. Hoover 6, considering methods for group‐administered treatment trial, noted that the methods generally applied to cluster randomised trials may not be entirely applicable as they assume homogeneity of the clustering effect between treatment arms. For cluster randomised trials, it can been shown that estimation is robust to heteroscedasticity for continuous outcome measures, both theoretically 3 and empirically 7, due to the expected distributions of cluster sizes being the same in each arm. In contrast, both cluster sizes and intra‐cluster correlation may differ systematically between arms in trials of group‐administered treatments or health professional activities, due to differences in the organisation and delivery of care between treatment arms, with a result in a loss of robustness for some estimation methods 3.

This paper is concerned with trials where a treatment with clustering is compared with a treatment without. This could be where a group‐administered treatment is compared with an un‐clustered treatment, such as drug treatment, treatment as usual or patient information. This trial design has been called a *partially* nested 4, 8, contrasting with a *fully* nested design of cluster randomised trials or trials comparing only clustered treatments. To simplify discourse, we will assume that the un‐nested arm is the control.

Several papers have considered the design and analysis for partially nested designs 3, 8, 9 for continuous outcome measures. The simplest of these methods is to compute summary measures for each cluster in the nested arm. These summary measures are then compared with the individual patient outcome data in the control arm. Because the variance of the summary measures will be smaller than the variance of the individuals in the control, Roberts and Roberts 3 recommended that summary measures analysis use the Satterthwaite test 10. Sometimes referred to as the unequal variance *t*‐test, this corrects for unequal variance by modifying the degrees of freedom of the *t*‐distribution. Where adjustment for baseline covariates is required, Roberts and Roberts 3 and Moerbeek and Wong 8 describe regression methods using a random effect for therapy group. Care needs to be taken when applying general methods for clustering to partially nested data as between‐arm heteroscedasticity may bias inference if not correctly modelled 3.

This paper evaluates statistical methods for partially nested binary data. While this design can occur in other settings, the motivation and focus of this paper are trials comparing group‐administered treatments with an un‐nested control. Moerbeek and Wong 8 briefly consider this issue suggesting a method for sample size calculation based on proportions and the design effect. In Section 2, we consider methods that might be used for partially nested binary data. In Section 3, we evaluate these methods in a simulation study considering consistency and small sample properties including bias and test size. Methods to calculate sample size and power are presented in Section 4, before comparing these with empirical power determined by simulation.

## 2 Methods for the analysis of partially nested data binary data

There is an extensive literature considering methods for the analysis of binary outcomes in cluster randomised trials; see, for example, Donner and Klar 11, Hayes and Moulton 12 or Eldridge and Kerry 13 with Donner *et al.* 14 discussing recent developments. A first group of methods considers statistical analysis on the scale of proportions including a summary measures test and a test of proportions with standard errors adjusted by a design effect term. A second group is based on logistic regression models including random effects models and generalised estimating equations. We will now consider the adaption of these to the partially nested trial design.

### 2.1 Summary measures test for a partially nested design

Consider a partially nested trial comparing a group therapy (*G*) causing clustering and an un‐nested control therapy (*C*) and let *N*_*G*_ and *N*_*C*_ be the total number of subjects in each treatment arm. Suppose *y_i_* is a binary outcome for the *i*th subject. In the group intervention arm, suppose there are *k* groups indexed by *j* with *j* being a function of *i*. Let *π*_*G*_ and *π*_*C*_ be the proportions of subjects with the event (*y_i_* = 1). Let *m_j_* be the number of subjects in the *j*th group so that $N_{G}=\sum_{j=1}^{k}m_{j}$. In the control arm, an unbiased estimator of *π*_*C*_ is *p*_*C*_ = *r*_*C*_/*N*_*C*_, where the number of subjects with the event is *r*_*C*_. Considering the group treatment arm, let *π*_*G*,*j*_ be the proportion of subjects with the event in the *j*th therapy group. Define *r*_*j*_ as the number of subjects with the event in the *j*th group and *q*_*j*_as the sample proportion estimator of *π*_*G*,*j*_, given by *q*_*j*_ = *r*_*j*_/*m*_*j*_. A summary measures estimator of *π*_*G*_ can be defined as $p_{G}=\frac{\sum_{j}^{k}q_{j}}{k}$ and the treatment effect estimated by *p*_*G*_ − *p*_*C*_. Assuming *p*_*G*_ and *p*_*C*_ are normally distributed, a test can be based on the statistics

(1) $$T=\frac{p_{G}-p_{C}}{SE\left[p_{G}-p_{C}\right]}=\frac{p_{G}-p_{C}}{\sqrt{SE{\left[p_{G}\right]}^{2}+SE{\left[p_{C}\right]}^{2}}}$$

with confidence interval *p*_*G*_ − *p*_*C*_ ± *z*_*α*/2_ *SE*[*p*_*G*_ + *p*_*C*_]. The term *SE*[*p*_*G*_] can be estimated by the standard error of the sample mean of the summary measures. Defining

(2) $$s_{G}=\sqrt{\frac{\sum_{j=1}^{k}{\left(q_{j}-p_{G}^{\text{sum}}\right)}^{2}}{\left(k-1\right)}}\text{,}$$

then

(3) $$SE\left[p_{G}\right]=\sqrt{\frac{s_{G}^{2}}{k}}=\sqrt{\frac{\sum_{j=1}^{k}{\left(q_{j}-p_{G}^{\text{sum}}\right)}^{2}}{\left(k-1\right)k}}\text{,}$$

which is a moment estimator. A commonly used estimator of *SE*[*p*_*C*_] is $\sqrt{\frac{p_{C}\left(1-p_{C}\right)}{N_{C}}}$, but this is a maximum likelihood estimate. To keep estimation methods consistent between arms, the sample standard deviation of the control arm observation could be used, which is

(4) $$s_{C}=\sqrt{\frac{\sum_{i=1}^{N_{c}}Y_{i}^{2}-N_{C}p_{C}^{2}}{\left(N_{C}-1\right)}}=\sqrt{\frac{N_{C}p_{C}-N_{C}p_{C}^{2}}{\left(N_{C}-1\right)}}=\sqrt{\frac{p_{C}\left(1-p_{C}\right)N_{C}}{\left(N_{C}-1\right)}}\text{.}$$

The moment‐based estimator of the control arm standard error is therefore

(5) $$SE\left[p_{C}\right]=\sqrt{\frac{s_{C}^{2}}{N_{C}}}=\sqrt{\frac{p_{C}\left(1-p_{C}\right)}{\left(N_{C}-1\right)}}$$

If the standard errors of *p*_*C*_ and *p*_*G*_ defined by (3) and (5) are used, Equation (1) is simply the test statistic of an unequal variance test of means applied to the binary observations in the control arm and the summary measures in the group arm.

### 2.2 Satterthwaite test

With a large sample of both groups in the clustered arm and subjects in the un‐clustered control, a normal approximation can be justified for a summary measures test, but where the numbers of groups is small, this is less tenable. Because $E\left[s_{G}^{2}\right]$ will generally be smaller than *p*_*C*_, this suggests a summary measures analysis based on the Satterthwaite test 10 using Equation (1) but assuming a *t*‐distribution with degrees of freedom defined by

(6) $$\upsilon =\frac{{\left(\frac{s_{G}^{2}}{k}+\frac{s_{C}^{2}}{N_{C}}\right)}^{2}}{\frac{{\left(\frac{s_{G}^{2}}{k}\right)}^{2}}{k-1}+\frac{{\left(\frac{s_{C}^{2}}{N_{C}}\right)}^{2}}{N_{C}-1}}$$

with $E\left[s_{G}^{2}\right]$ and $s_{C}^{2}$ defined by Equations (2) and (4).

### 2.3 Adjusted test of proportions

The two‐sample *z*‐test of proportions without clustering has a null standard error

$$SE_{\text{null}}\left[p_{G}-p_{C}\right]=\sqrt{p\left(1-p\right)\left(\frac{1}{N_{G}}+\frac{1}{N_{C}}\right)}\text{,}$$

which was used for hypothesis testing where *p* is the pooled proportion, and a non‐null standard error

$$SE\left[p_{G}-p_{C}\right]=\sqrt{\frac{p_{G}\left(1-p_{G}\right)}{N_{G}}+\frac{p_{C}\left(1-p_{C}\right)}{N_{C}}}\text{,}$$

used for confidence interval construction. Assuming normality, this gives a test statistic $T=\frac{p_{G}-p_{C}}{SE_{\text{null}}\left[p_{G}-p_{C}\right]}$ and a (1 − *α*) confidence interval given by *p*_*G*_ − *p*_*C*_ ± *z*_*α*/2_ *SE*[*p*_*G*_ − *p*_*C*_].

Considering now a partially nested design, one can define a weighted estimator of the treatment effect as

$$p_{G}-p_{C}=\frac{\sum_{j}w_{j}q_{j}}{\sum_{j}w_{j}}-p_{C}\text{.}$$

The standard choices are (i) to weight each cluster equally (*w*_*j*_ = 1) giving the summary measures estimate, (ii) to weight according to the cluster size (*w*_*j*_ = *m*_*j*_) or (iii) to use a minimum variance weighting $\left(w_{j}=\left(\frac{m_{j}}{1+\left(m_{j}-1\right)\rho }\right)\right)$ where *ρ* is an estimate of the intra‐cluster correlation of the clustering effect, which can be estimated using analysis of variance (see, for example, Donner 11 (page 84)).

The variance of *p*_*C*_ is $Var\left[p_{C}\right]=\frac{\pi_{C}\left(1-\pi_{C}\right)}{N_{C}}$. From Jung *et al.* 15, $Var\left[p_{G}\right]=\frac{\pi_{G}\left(1-\pi_{G}\right)}{N_{G}}D$ with *D* depending on the choice of weights. Where clusters are weighted equally, $D=\bar{m}\left(\rho +\frac{\left(1-\rho \right)}{k}\sum_{j}\frac{1}{m_{j}}\right)$ with $\bar{m}$ being the mean cluster size $\frac{\sum_{j}m_{j}}{k}$.

Where clusters are weighted by cluster size (*m_j_*), $D=1+\left(\frac{\sum_{j}m_{j}^{2}}{\sum_{j}m_{j}}-1\right)\rho$.

If minimum variance weights are applied, $D=\frac{N_{G}}{\sum_{j}\frac{m_{j}}{1+\left(m_{j}-1\right)\rho }}$.

Replacing *π*_*C*_ and *π*_*G*_ by their estimators *p*_*C*_ and *p*_*G*_, one obtains the standard error estimates $SE_{\text{null}}\left[p_{G}-p_{C}\right]=\sqrt{p\left(1-p\right)\left(\frac{D}{N_{G}}+\frac{1}{N_{C}}\right)}$ and $SE\left[p_{G}-p_{C}\right]=\sqrt{\frac{p_{G}\left(1-p_{G}\right)}{N_{G}}D+\frac{p_{C}\left(1-p_{C}\right)}{N_{C}}}$.

A normal based test statistic is therefore given by $T=\frac{p_{G}-p_{C}}{SE_{\text{null}}\left[p_{G}-p_{C}\right]}$ with a (1 − *α*) confidence interval: *p*_*G*_ − *p*_*C*_ ± *z*_*α*/2_ *SE*[*p*_*G*_ − *p*_*C*_]. One should note that the uncertainty in the estimation *SE*[*p*_*G*_ − *p*_*C*_] has been ignored, in contrast to the summary measures procedure based on the Satterthwaite test.

### 2.4 Logistic random effect models

Consider now a logistic random effects model with a random effect *u*_*j*_ to model the between‐cluster variation in the group intervention arm. The distribution of the random effect is generally taken to be $N\left[0,\sigma_{U}^{2}\right]$. With*y*_*i*_ ~*Bernouli*[*π*_*i*_], two parameterizations might be considered, a logistic random intercept (LRI) given by

(7) logitπi=α+βxi+Iδi+uj

and a logistic random coefficient (LRC) model

(8) logitπi=α+βxi+Iδi+Iuji

In either model, *I*_*i*_ is an indicator variable for the group‐administered treatment arm and *δ* is the treatment effect on the log‐odds scale. Inference on *δ* can be based on a Wald test or a likelihood ratio test. It should be noted also that the log‐likelihoods of models based on Equations (7) and (8) are equal for this design, but this does not hold for the null model in which *δ* = 0. For both models, the intra‐class correlation on the log‐odds scale is defined as $\rho_{L}=\frac{\sigma_{U}^{2}}{\sigma_{U}^{2}+\frac{\pi^{2}}{3}}$ 16, where for this formula, *π* is the mathematical constant. Where clusters vary in size, random effects models apply minimum variance weighting.

In general, for a logistic‐normal model, the marginal probability of positive outcome, say *π*_1_, for a given value of the linear predictor *η*, is

$$\pi_{1}=\mathrm{Pr}\left[y_{i}=1\right]=\int_{-\infty }^{+\infty }\frac{\exp\left(\eta +z\sigma_{U}\right)}{1+\exp\left(\eta +z\sigma_{U}\right)}\phi \left(z\right)dz\text{,}$$

where *ϕ*(*z*) is the standard normal density. The joint probability of two positive outcomes in the same cluster, say*π*_11_, is defined as

$$\pi_{11}=\mathrm{Pr}\left[y_{ij}=1,y_{i^{\prime }j}=1\right]=\int_{-\infty }^{+\infty }{\left\{\frac{\exp\left(\eta +z\sigma_{U}\right)}{1+\exp\left(\eta +z\sigma_{U}\right)}\right\}}^{2}\phi \left(z\right)dz\;,\;for\;i\neq i^{\prime }\text{.}$$

Both *π*_1_ and *π*_11_ can be determined from model estimates using numerical integration (see Rodriguez and Elo 17 for a stata 18 algorithm (*xtrhoi*) for this purpose). The intra‐cluster correlation on the manifest scale is then given by $\rho =\frac{\pi_{11}-\pi_{1}^{2}}{\pi_{1}\left(1-\pi_{1}\right)}$.

For both models (7) and (8), the marginal probability of positive outcome *π*_*G*_ is

$$\pi_{G}=\mathrm{Pr}\left(y_{i}=1\right)=\int_{-\infty }^{+\infty }\frac{\exp\left(\alpha +\delta +z\sigma_{U}\right)}{1+\exp\left(\alpha +\delta +z\sigma_{U}\right)}\phi \left(z\right)dz$$

in the intervention arm. Under model equations (7) and (8),

$$\pi_{C}=\mathrm{Pr}\left(y_{i}=1\right)=\int_{-\infty }^{+\infty }\frac{\exp\left(\alpha +z\sigma_{U}\right)}{1+\exp\left(\alpha +z\sigma_{U}\right)}\phi \left(z\right)dz$$

Hence, for model (7),

$$\pi_{G}-\pi_{C}=\int_{-\infty }^{+\infty }\frac{\exp\left(\alpha +\delta +z\sigma_{U}\right)}{1+\exp\left(\alpha +\delta +z\sigma_{U}\right)}\phi \left(z\right)dz-\int_{-\infty }^{+\infty }\frac{\exp\left(\alpha +z\sigma_{U}\right)}{1+\exp\left(\alpha +z\sigma_{U}\right)}\phi \left(z\right)dz$$

so *δ* = 0 implies *π*_*G*_ equals *π*_*C*_. A null treatment effect in the random intercept model equates to a null effect on the scale of proportions. In model (8),

$$\pi_{C}=\mathrm{Pr}\left(y_{i}=1\right)=\frac{\exp\left(\alpha \right)}{1+\exp\left(\alpha \right)}\text{.}$$

When *δ* = 0,

(9) $$\pi_{G}-\pi_{C}=\int_{-\infty }^{+\infty }\frac{\exp\left(\alpha +z\sigma_{U}\right)}{1+\exp\left(\alpha +z\sigma_{U}\right)}\phi \left(z\right)dz-\frac{\exp\left(\alpha \right)}{1+\exp\left(\alpha \right)}\text{,}$$

which is non‐zero, unless *α* = 0 which corresponds to *π*_*G*_ = *π*_*C*_ = 0.5 or if trivially $\sigma_{u}^{2}=0$. Hence, a null effect in model (8), that is, *δ* = 0, does not imply *π*_*G*_ equals *π*_*C*_. The magnitude of (9) will increase as the value $\sigma_{u}^{2}$ increases. Thus, a random coefficient model will not be consistent with a null effect on the scale of proportions. It can also be seen from Equation (9) that this effect does not depend on cluster size. We can summarise this as follows: the random intercept model estimates a cluster‐specific effect in both arms whereas the random coefficient model estimates a cluster‐specific effect in the clustered arm and a marginal effect in the comparator. This suggests that the random coefficient model (LRC) defined by Equation (8) may have a substantial bias for a null treatment effect on the scale of proportions, a hypothesis that will be considered in the succeeding text in Section 3.2.

### 2.5 Logistic generalised estimating equation models

An alternative model‐based analysis is the method of generalised estimating equations 19. This can be applied with a logistic link function 13. For partially nested binary data, the model is given by

logitπi=α+βxi+Iδi,

where a logistic link function is used. The treatment effect estimate *δ* is the marginal or population‐averaged log‐odds. Inference on the treatment effect controlling for the clustering effect can be carried using robust standard errors 20. The choice of working correlation matrix affects the weighting of subjects. Use of an identity matrix weights subjects equally, whereas an exchangeable working correlation matrix applies minimum variance weights. Provided there is a clustering effect $\left(\sigma_{u}^{2}>0\right)$, the population‐averaged log‐odds ratio will be smaller in magnitude than the cluster‐specific estimate of the log‐odds ratio derived from the random effects models in the previous text.

## 3 Consistency and small sample bias

In clinical trials without clustering, tests of proportions and analyses based on logistic regression models are consistent for a null treatment effect, even where baseline covariates are included in the logistic model (Robinson and Jewel 21). The same property is desirable when selecting methods of analysis for partially nested trials, as without this, one could have a treatment effect on one scale of estimation or method of analysis but not on another. In Section 2.4, we saw that treatment effect estimates from the LRC model was not consistent for a null effect on the scale of proportions. We therefore begin the evaluation of methods by checking consistency for a null effect. We then considered small sample properties including bias for estimates of the intra‐cluster correlation and the treatment effect for the null and non‐null cases, and for test size. In doing this, the objective was to check the effect of cluster size, intra‐cluster correlation and the event rate on the performance of the analysis methods.

### 3.1 Simulating partially nested binary data

Simulation studies examining the performance of methods for clustered binary data often use the beta‐binomial model (see, for example, Jung *et al.* 15, Bellamy *et al.* 22 Lee 23, Ukoumunne *et al.* 24, Austen 25 and Ma *et al.* 26), which is convenient because the beta‐binomial distribution can be parameterized in terms of *π* and the manifest intra‐cluster correlation coefficient (*ρ*). We chose instead to use the logistic‐normal distribution to avoid possible artefacts due to differences between the random effect distributions of data generation and method of analysis. Unlike the beta‐binomial, data generation using a logistic‐normal distribution allows covariates to be added to the linear predictor, although investigation of covariate adjustment is beyond the scope of this paper. For this data generation model, one option is to base simulation scenarios on values of random effects variance, *σ*_*U*_ 27, 28, 29, 30. We consider this to have disadvantages as it is the proportions *π*_*G*_ and *π*_*C*_, and the manifest intra‐cluster correlation (*ρ*), which are usually considered when designing such a study. Thus, for specified values of *π*_*G*_ and *ρ*, we determined the values *η*_*G*_ and *σ*_*U*_ such that

(10) $$\pi_{G}=\int_{-\infty }^{+\infty }\frac{\exp\left(\eta_{G}+z\sigma_{U}\right)}{1+\exp\left(\eta_{G}+z\sigma_{U}\right)}\phi \left(z\right)dz$$

(11) $$\text{and}\;\rho =\frac{\int_{-\infty }^{+\infty }{\left(\frac{\exp\left(\eta_{G}+z\sigma_{U}\right)}{1+\exp\left(\eta_{G}+z\sigma_{U}\right)}\right)}^{2}\phi \left(z\right)dz-\pi_{G}^{2}}{\pi_{G}\left(1-\pi_{G}\right)}\text{.}$$

For each cluster *j*, a value *η*_*j*_ was sampled from a normal distribution $N\left[\eta_{G},\sigma_{U}^{2}\right]$. We then generated the binary outcome using a Bernoulli distribution with parameter *π*_*j*_ = exp(*η*_*j*_)/(1 + exp(*η*_*j*_)) for the *j*th cluster in the group arm and *π*_*C*_ in the control arm. All simulation work was carried out using stata 18.

Treatment therapy groups used in healthcare are generally quite small. The desirable group size is often specified in the treatment manual, and so one would expect the coefficient of variation (c.v.) of the distribution of group sizes to be quite small. For example, in a trial of a group‐based cognitive behavioural intervention for persistent lower back pain, the mean group size was 5.0 (standard deviation (s.d.) = 1.5, c.v. = 0.3) 31. A class‐based programme for treatment of knee osteoarthritis had groups with mean size 10.1 (s.d. = 2.59, c.v. = 0.26) 32. In a trial of group cognitive behavioral therapy for patients with schizophrenia, the mean group size was 5.7 (s.d. = 0.48, c.v. = 0.09) 33. We have therefore focused on the situation were cluster sizes are between 5 and 10.

The cluster randomised trial literature demonstrates that the impact of cluster size variation depends on the weighting of subjects by the analysis method 34. Where the analysis methods weight subjects equally, the design effect is a linear function of the square of the c.v. 35. Where the analysis applies minimum variance weights, the situation is more complex with the design effect being dependent on the distribution of cluster sizes 36. Perhaps for this reason, studies evaluating the effects of cluster size variation have generally considered just a single method of analysis. Given that different methods of analyses weight subjects in different ways, it would be quite complex to incorporate cluster size variation into a comparison of the methods of analyses weighting subjects in different ways. For clarity of exposition, we have therefore chosen to assume equal cluster sizes. The implications of cluster size variation for power of this study design are discussed in Section 4.3 in the succeeding text.

Estimated intra‐cluster correlation coefficients obtained from trials are generally imprecise. For example, Barrowclough *et al.* 33 report 30 estimates of the intra‐cluster correlation coefficient for group CBT with estimates ranging from 0 (95% CI 0 to 0.29) to a maximum of 0.26 (95% CI 0.02 to 0.67) with a mean of 0.044. There is uncertainty regarding plausible population value of intra‐cluster correlation coefficient on which to base a simulation study. This can also be an issue when considering the magnitude of the treatment effect when designing trials more generally. In the absence of reliable information regarding the treatment effect, one approach is to use a Cohen effect size. By expressing the treatment effect as a variance, it can be shown that medium (0.5) and large (0.8) Cohen effect sizes correspond to 5.9% and 13.8% of the total variance. While this is related to intervention effects, it suggests 0.05 and 0.1 to be plausible population values of the manifest intra‐cluster correlation coefficient, when considering clustering due to treatment.

### 3.2 Consistency for a null treatment effect

To estimate the asymptotic value of a null treatment effect, a single dataset with a large sample size was simulated using the methods described in the previous text. We considered *π* = *π*_*G*_ = *π*_*C*_ taking values (0.05, 0.1, 0.2, 0.3, 0.4, 0.5, 0.6, 0.7, 0.8, 0.9, 0.95), the manifest intra‐class correlation coefficient (*ρ*) equal to 0, 0.05 or 0.1, and the cluster size (*m*) in the nested arm equal to 5, 10 and 20.

The combination of simulation parameters that would be expected to give the least precise estimate of the log‐odds ratio of the treatment effect were *π* equal to 0.05 or 0.95, intra‐cluster correlation coefficient *ρ* = 0.1 and a cluster size (*m*) of 20. Based on pilot work, we estimated that a sample size of 400 000 in each arm would give a 95% confidence interval for a null treatment effect on the scale of the log‐odds ratio for this model of width 0.1. As equal size clusters have been used, the summary measures test (SMT), the Satterthwaite test (SATT) and the adjusted test of proportions (ATP) have the same point estimates.

Table 1 shows the 95% confidence interval for the null treatment effect for all methods for a cluster size of 10. Values where the lower confidence limit is above zero or the upper confidence limit is below zero have been emboldened. Where the data‐generating ICC is non‐zero, the confidence intervals for the LRC model exclude zero unless *π*_*G*_ = *π*_*C*_ = 0.5. For other methods of analysis, there are just two instances where the confidence interval does not include zero that can be readily explained by sampling variation.

**Table 1:** Confidence interval for the null treatment effect  (*π* = *π*_*G*_ = *π*_*C*_) with a total sample size of 800 000 for various methods of analysis (40 000 clusters of size 10 vs. 400 000 controls).

ICC	*π*	Adjusted test of proportions/summary measure test (ATP/SMT)	Logistic GEE (LGEE)	Logistic random intercept (LRI)	Logistic random coefficient (LRC)
		95% CI	95% CI	95% CI	95% CI
0	0.05	(−0.002, 0.000)	(−0.037, 0.003)	(−0.037, 0.003)	(−0.048, 0.000)
	0.1	(−0.002, 0.000)	(−0.025, 0.004)	(−0.025, 0.004)	(−0.025, 0.004)
	0.2	(−0.001, 0.002)	(−0.006, 0.016)	(−0.006, 0.016)	(−0.006, 0.016)
	0.3	(−0.002, 0.002)	(−0.011, 0.008)	(−0.011, 0.008)	(−0.011, 0.008)
	0.4	(**0**.**000**, 0.004)	(−0.001, 0.017)	(−0.001, 0.017)	(−0.002, 0.016)
	0.5	(−0.005, 0.000)	(−0.018, −0.001)	(−0.018, −0.001)	(−0.018, −0.001)
	0.6	(−0.002, 0.002)	(−0.009, 0.008)	(−0.009, 0.008)	(−0.009, 0.008)
	0.7	(−0.001, 0.003)	(−0.005, 0.014)	(−0.005, 0.014)	(−0.005, 0.014)
	0.8	(−0.001, 0.002)	(−0.006, 0.015)	(−0.006, 0.015)	(−0.006, 0.015)
	0.9	(−0.001, 0.002)	(−0.010, 0.019)	(−0.010, 0.019)	(−0.010, 0.022)
	0.95	(−0.001, 0.001)	(−0.020, 0.020)	(−0.020, 0.020)	(−0.018, 0.030)
0.05	0.05	(−0.002, 0.000)	(−0.035, 0.009)	(−0.037, 0.010)	(−0.419, −**0**.**362**)
	0.1	(−0.002, 0.000)	(−0.027, 0.005)	(−0.028, 0.006)	(−0.232, −**0**.**194**)
	0.2	(−0.002, 0.002)	(−0.012, 0.012)	(−0.013, 0.013)	(−0.105, −**0**.**079**)
	0.3	(−0.002, 0.002)	(−0.011, 0.010)	(−0.011, 0.011)	(−0.059, −**0**.**037**)
	0.4	(−0.001, 0.004)	(−0.004, 0.016)	(−0.004, 0.017)	(−0.025, −**0**.**005**)
	0.5	(−0.002, 0.003)	(−0.008, 0.011)	(−0.009, 0.012)	(−0.009, 0.011)
	0.6	(−0.001, 0.003)	(−0.006, 0.014)	(−0.006, 0.015)	(**0**.**016**, 0.036)
	0.7	(−0.003, 0.002)	(−0.013, 0.009)	(−0.013, 0.009)	(**0**.**034**, 0.056)
	0.8	(−0.003, 0.001)	(−0.016, 0.008)	(−0.017, 0.008)	(**0**.**076**, 0.102)
	0.9	(−0.001, 0.002)	(−0.014, 0.018)	(−0.015, 0.019)	(**0**.**181**, 0.218)
	0.95	(−0.001, 0.001)	(−0.023, 0.021)	(−0.024, 0.023)	(**0**.**347**, 0.404)
0.1	0.05	(−0.001, 0.001)	(−0.031, 0.018)	(−0.034, 0.020)	(−0.728, −**0**.**660**)
	0.1	(−0.003, 0.000)	(−0.032, 0.003)	(−0.036, 0.003)	(−0.430, −**0**.**387**)
	0.2	(−0.002, 0.002)	(−0.012, 0.014)	(−0.014, 0.016)	(−0.209, −**0**.**179**)
	0.3	(−0.004, 0.001)	(−0.019, 0.004)	(−0.021, 0.005)	(−0.124, −**0**.**099**)
	0.4	(−0.006, 0.000)	(−0.023, −0.002)	(−0.026, −0.002)	(−0.071, −**0**.**048**)
	0.5	(−0.002, 0.003)	(−0.008, 0.013)	(−0.009, 0.014)	(−0.009, 0.014)
	0.6	(**0**.**000**, 0.005)	(−0.002, 0.020)	(−0.002, 0.022)	(**0**.**043**, 0.066)
	0.7	(−0.004, 0.001)	(−0.019, 0.004)	(−0.021, 0.005)	(**0**.**084**, 0.109)
	0.8	(−0.003, 0.001)	(−0.021, 0.006)	(−0.023, 0.006)	(**0**.**175**, 0.205)
	0.9	(−0.002, 0.001)	(−0.019, 0.016)	(−0.022, 0.018)	(**0**.**380**, 0.423)
	0.95	(−0.001, 0.001)	(−0.025, 0.023)	(−0.028, 0.026)	(**0**.**635**, 0.702)

P is the proportion.

GEE, generalised estimating equations.

**Figure 1:**
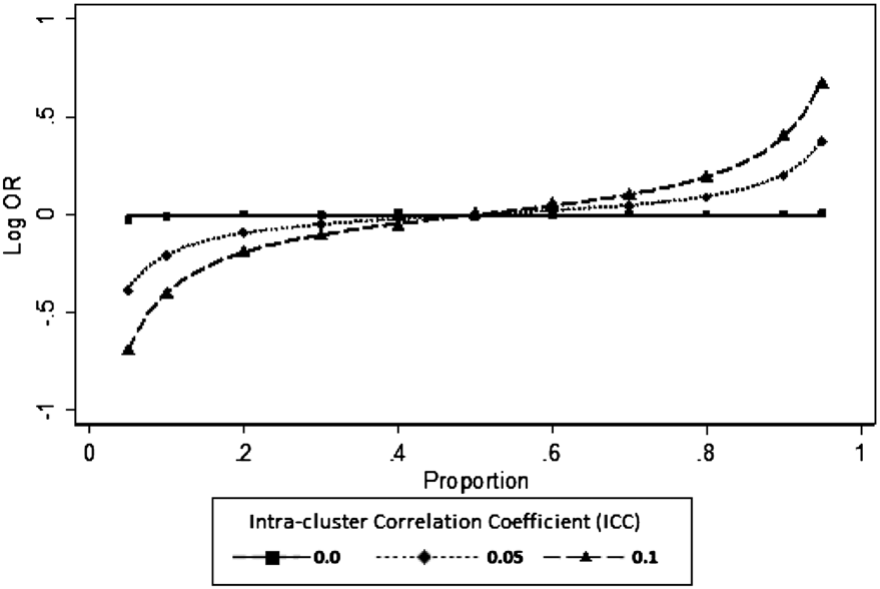
Bias in partially nested random coefficient model for the null case as a function of the proportion *π*_*G*_ > *π*_*C*_ > 0.5. Line constructed by numerical integration. Points by simulation using 40 000 clusters of size 10 and 400 000 controls. OR, odds ratio.

Figure 1 plots the estimate of treatment effect log‐odds ratio against *π* for the LRC model for a cluster size of 10. Corresponding values were obtained using numerical integration of Equation (9) for (*ρ*) equal to 0, 0.05 or 0.1 with *π* = *π*_*G*_ = *π*_*C*_ over the range [0.05, 0.95] at 0.025 intervals shown as lines. It can be seen that values obtained by simulation and by numerical integration are very close. As *π* departs from 0.5, the bias increases, and this become large when *π* approaches 0 or 1. We have confirmed empirically that the bias suggested in Section 2.4 is present and can be substantial. For this reason, we do not recommend the use of model (8). Almost identical tables and figures were obtained for cluster sizes of 5 and 20 and so these are not presented.

The ATP, the logistic random effect models (LRI and LRC) and the logistic generalised estimation equations (LGEE) model all give estimates of the intra‐cluster correlation coefficient, *ρ*. In the very large samples considered here, estimates had minimal bias for *ρ* for all the estimation methods. The largest absolute deviation from the data‐generating value was 0.0065 for the ATP methods occurring when *π* = 0.05, *m* = 5, *ρ* = 0.1 (data not shown), suggesting all methods were consistent.

### 3.3 Small sample properties

A second simulation study investigated the small sample properties of estimators. The objective was to assess possible biases in the estimation of the intra‐cluster correlation coefficient of the clustered arm, the treatment effect and the test size. Five methods of analysis were compared, namely the ATP estimator, the summary measures estimator (SMT), the SATT, the LGEE model and the random intercept model (LRI). To simplify interpretation, the design was balanced in terms of cluster size, study size and intra‐cluster correlation coefficient. We considered the following partially nested designs with the same number of controls subjects as in the clustered arm: (i) 20 clusters of 5, (ii) 10 clusters of 10 subjects, (iii) 40 clusters of 5, (iv) 20 clusters of 10, (v) 80 clusters of 5 and (vi) 40 clusters of 10. For each of these, we considered combinations of *π*_*G*_, *π*_*C*_ ∈ {0.1, 0.2,.., 0.9} and the manifest intra‐cluster correlation coefficient *ρ* equal to 0, 0.05 or 0.1. To restrict the size of the simulation study and simplify presentation of results, pairs of *π*_*C*_ and *π*_*G*_ where chosen that gave a log‐odds ratio for the treatment effect of magnitude less than 1.5, as performance in situations where it is larger is of less concern. This reduced the number of simulation scenarios from a possible 1458 (3 × 2 × 3 × 81) to 846 (3 × 2 × 3 × 47). For each scenario, 20 000 simulations were carried out as this would give 95% confidence intervals of the test size of width less than ± 0.005 where the empirical test size is below 0.07.

There were 210 failures in the 16 920 000 simulations (0.0013%). Of these failures, 162 occurred when the sample size in each arm was 100. Ninety‐eight of these failures related to logistic models occurring when the observed event rate was 0 or 1 in either the control or group arm.

**Figure 2:**
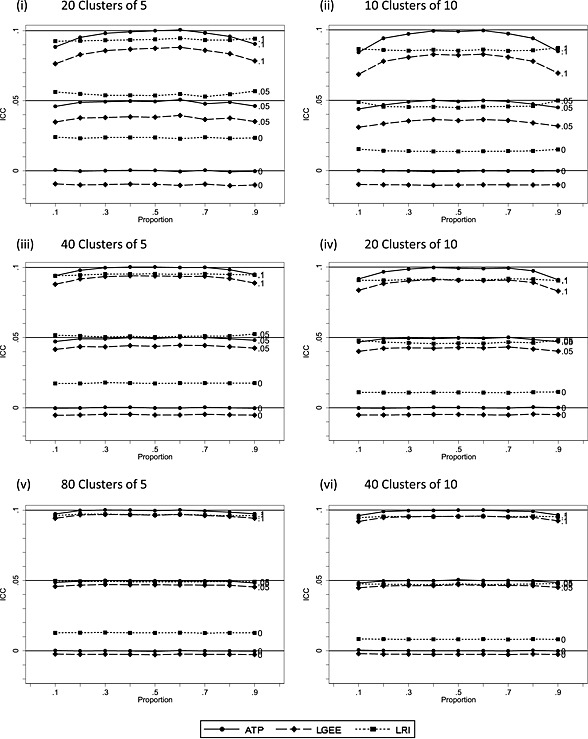
Estimates of the intra‐cluster correlation coefficient for the adjusted test of proportions (ATP), logistic generalised estimating equations (LGEE) and logistic random intercept (LRI) models for small samples as a function of the proportion *λ* ∈ (1, *λ*_*eq*_). Control arm has the same number of subjects with no clustering. Three values of the ICC are considered as indicated by the numeric labels on each line.

Figure 2 displays the mean values of estimates of the intra‐cluster correlation coefficients against prevalence, obtained from the ATP, the generalised estimating equations model (LGEE) and the LRI model methods, for the null case (*π_C_* = *π_G_*). The analysis of variance‐based estimate, used in the adjusted test of proportions, is closest to the data‐generating values of *ρ* when the event rate was 0.5. The LGEE method underestimated for all three data‐generating values of *ρ*, but this bias is much smaller for larger sample sizes. The LRI model overestimates the zero value. This effect is greater for the smaller cluster size of 5 and can be explained by the random effect variance being constrained to be non‐negative. As the data‐generating values of *ρ* increased, this positive bias for LRI reduced in all sample sizes. When *ρ* was 0.1, there is a downward bias, as one might expect for a maximum likelihood estimate. Similar patterns of underestimation or overestimation are also observed in the non‐null case (not presented). The effect of underestimation or overestimation of *ρ* or the related variance component will tend to make inference respectively more or less conservative.

For the treatment effect, bias has been estimated as the difference between the mean of the simulated estimates and the data‐generating value. For the adjusted tests of proportions, summary measures and the Satterthwaite test, this is a difference between the mean of estimates and (*π*_*G*_ − *π*_*C*_). For the logistic generalised estimating methods (LGEE), the bias is calculated as the difference between the mean of estimates of the log‐odds ratio and the data‐generating marginal log‐odds ratio (log_*e*_[(*π*_*G*_/(1 − *π*_*G*_))/(*π*_*C*_/(1 − *π*_*C*_))]). For the LRI model, the bias is calculated as the difference between the mean of the cluster‐specific log‐odds ratio estimates and (*η*_*G*_ − *η*_*C*_), where *η*_*G*_ is the data‐generating values defined in the previous text and *ρ* is the solution of $\int_{-\infty }^{+\infty }\frac{\exp\left(\eta_{C}+z\sigma_{U}\right)}{1+\exp\left(\eta_{C}+z\sigma_{U}\right)}\phi \left(z\right)dz=\pi_{C}$. For each estimate of bias, a Monte‐Carlo confidence interval was determined using the standard deviation of the simulation estimates.

As before, the adjusted tests of proportions, summary measures and the Satterthwaite test methods have the same treatment effect estimate. The Monte‐Carlo confidence interval of the bias failed to include zero in only 3.7% (6/162) of the null cases and 3.5% (24/ 684) of the non‐null case. As one might expect, there is no evidence of bias and so no further analysis is presented.

**Figure 3:**
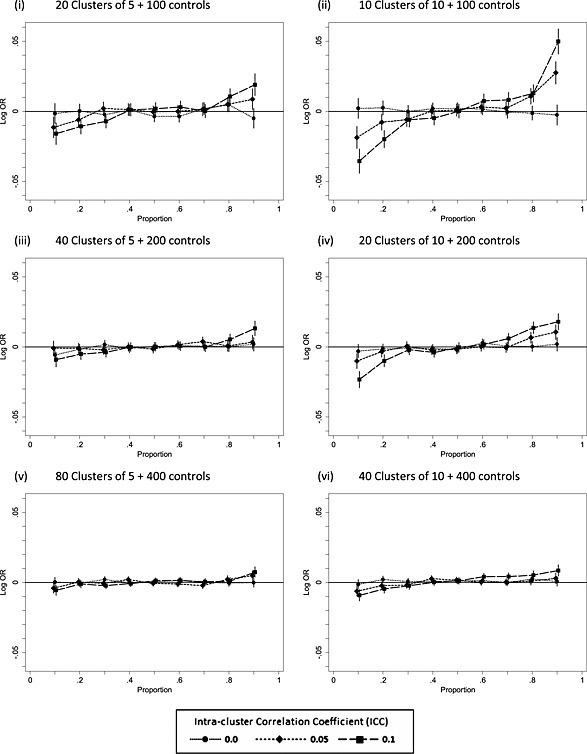
Bias in the treatment effect log‐odds ratio estimate under the null (*π_C_* = *π_G_*) as a function of the proportion*π*_*G*_ = *π*_*C*_ for the logistic generalised estimating equations model. OR, odds ratio.

Figure 3 plots the bias of the log‐odds ratio with Monte‐Carlo confidence interval for the LGEE method in the null case (log‐odds ratio = 0). Where the intra‐cluster correlation is non‐zero, the log‐odds ratio is negatively biased for *π*_*G*_ = *π*_*C*_ < 0.5 and positively biased for *π*_*G*_ = *π*_*C*_ > 0.5. These biases decrease with increased sample size and so do not contradict the results for consistency of this method. It should be noted also that the bias observed here, although similar in shape to that for non‐consistency for the LRC model (Figure 1 and Table 1), is substantially smaller and depends on cluster size with a size of 5 giving less bias than that of 10. Note also that the bias is greater where there are 20 clusters of size 10 (Figure 3(iv)) as compared with 20 clusters of size 5 (Figure 3(i)).

**Figure 4:**
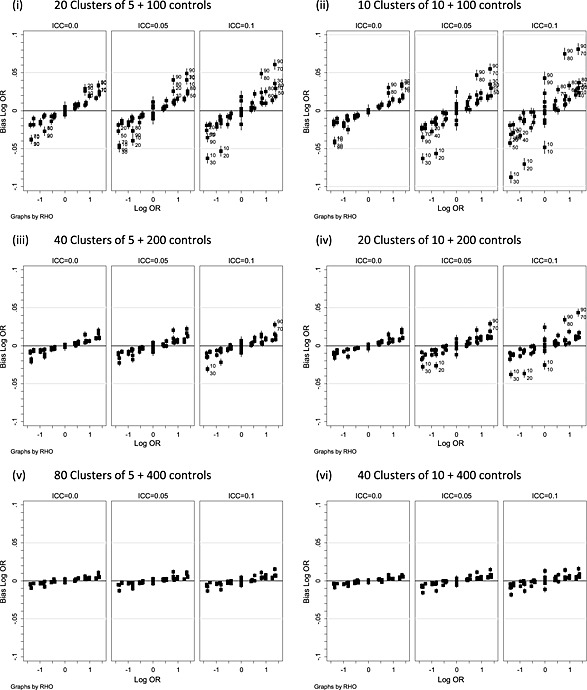
Bias in the treatment effect log‐odds ratio estimate for logistic generalised estimating equations (LGEE) model plotted against the data‐generating value for ICC(rho) = 0, 0.05 and 0.1. Data‐generating values of *π_G_* (superscript) *and π_C_* (subscript) are given where bias is greater than 0.025. OR, odds ratio.

Figure 4 considers the non‐null situation, plotting the bias with 95% Monte‐Carlo confidence interval of the bias against the data generation log‐odds ratio. Data‐generating values of *π_G_* (superscript) and *π_C_* (subscript) have been added to points where the magnitude of the bias is greater than 0.025. Among the 684 simulations for which the data‐generating log‐odds ratio was non‐zero, the bias is away from zero in almost all scenarios (664/684) and is greatest where the log‐odds ratio is further from zero. Of particular note is the bias when the data‐generating value of the ICC was zero as this suggests a bias for the non‐null case due to the method of estimation rather than data generation. As the sample size increases, the bias decreases as would be expected for a consistent estimator.

Under the null, results for the logistic random intercept model (Figure S1) are similar to the LGEE model (Figure 3). The non‐null (Figure S2) reveals a similar pattern of biases to the LGEE model (Figure 4) where the ICC is 0.05 or 0.1, but when the data‐generating ICC is 0, the bias is more marked than LGEE, and this is greater with a cluster size of 5 than 10. What is more, it is larger when the ICC is 0 than when the ICC is 0.05 or 0.1. This disparity can be explained by the substantial upward bias of the estimated ICC when the data‐generating value is zero (Figure 2). When the data‐generating ICC is 0, the data‐generating log‐odds are simply the log‐odds without clustering. In contrast, the estimated log‐odds will be the larger cluster‐specific value arising from the biased ICC estimate.

The presence of small sample bias for the treatment effect in the logistic models has implications for test size in each tail. Assuming no counteracting asymmetry in the standard error, the test size will increase for the tail in the direction of the bias.

It is generally recommended that two‐sided tests are carried out; see, for example, Bland and Altman 37. This recommendation provides protection against analysis bias due to post‐hoc selection of the more favourable one‐sided test. One might suppose therefore that test performance on each side is of no concern, but we disagree. If one rejects a null hypothesis of no treatment effect with a two‐sided test, one does not just conclude the treatments are different but draws inference that one treatment is better or worse than the other. In effect, a two‐sided test is a composite of two one‐sided tests. In rejecting the hypothesis in one or other direction, we implicitly presume that two 2.5% one‐sided tests have been carried out having a family‐wise error of 5%. In some settings, symmetry arguments allow us to conclude that test performance will be the same in either direction on theoretical grounds. Where there is between‐arm asymmetry in the design, as we have here, the assumption that two‐sided test performance accurately reflects the performance of either side may not be justified. We need therefore to consider test size for each side separately. For *z*‐ or *t*‐distributed test statistics, estimation of empirical test size of one‐sided tests is straightforward. For the likelihood ratio test based on a chi‐squared test, we have split the empirical test size according to whether the treatment effect log‐odds ratio was positive or negative.

**Figure 5:**
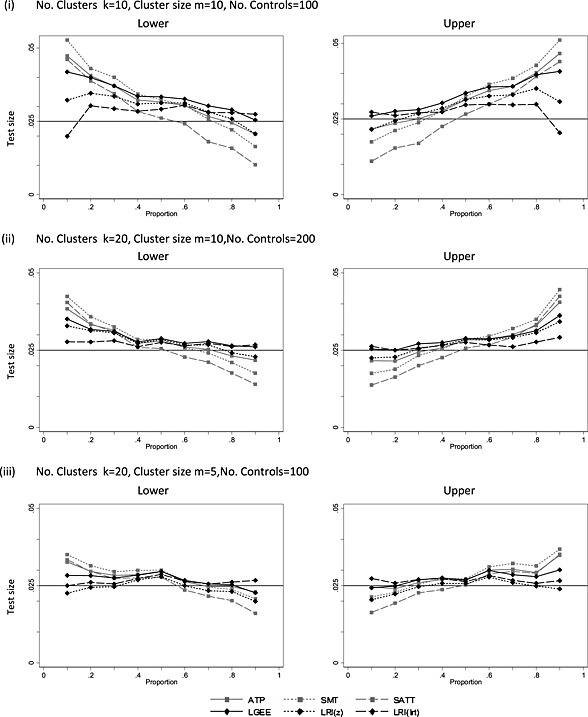
Empirical test size for lower and upper single‐sided (nominal 2.5% level) tests with ICC = 0.1 for the adjusted test of proportions (ATP), summary measures test (SMT), Satterthwaite *t*‐test (SATT), logistic generalised estimating equations (LGEE) model and a logistic random intercept model using a Wald test (LRI (*z*)) and a likelihood ratio test (LRI (lrt)) against null proportion.

Results for test size are presented in full for both two‐sided and one‐sided tests in Tables S1–S6. For all methods, the empirical two‐sided test size increases as *ρ* increases. Test size is consistently below the nominal level for the logistic random intercept model when *ρ* is zero, which can be explained by the upward bias on the estimated of *ρ* in this model (Figure 2). Considering now the one‐sided test results, the treatment effect bias observed for LGEE (Figure 3) and LRI (Figure S1) would be expected to increase the lower tail test size where *π*_*G*_ = *π*_*C*_ < 0.5 and increase the upper tail test size where *π*_*G*_ = *π*_*C*_ > 0.5. Figure 5 illustrates the test size for three scenarios from Tables S1–S6. As expected, lower tail test size is increased where *π*_*G*_ = *π*_*C*_ < 0.5 and upper tail test size is increased where *π*_*G*_ = *π*_*C*_ > 0.5 for most methods. Interestingly, the effect is more marked for ATP, SMT and SATT, for which there is no treatment effect bias. One explanation for bias of these methods is that they do not benefit from the variance stabilising effect of the logistic transformation, which should be important where *π*_*G*_ = *π*_*C*_ are close to either 0 or 1. The effect is most marked in the scenario with 10 clusters of size 10 and *π*_*G*_ = *π*_*C*_ = 0.1 or 0.9 (Figure 5(i)). Whereas the empirical two‐sided test size is close to the nominal value for the Satterthwaite test (Table S2), the size of the upper and lower tails are very different, with the type I error on one side being four times the type I error on the other side when *π*_*G*_ = *π*_*C*_ = 0.1 or 0.9. The asymmetry is larger for an ICC of 0.1 than 0.05 and is still present in larger sample sizes but to a lesser degree (Tables S5 and S6). Comparing results for clusters of size 10 and 5, the effect would also appear to be more marked where the cluster size is larger. For example, the scenario of 20 cluster of 10 (Figure 5(ii)) shows greater asymmetry than 20 of 5 (Figure 5(iii)) despite having twice the sample size. In conclusion, test size in a partially nested design with a binary outcome test size can be very different between sides, with the effect being greater where the event rates are closer to either 0 or 1, and also for larger cluster size and intra‐cluster correlation.

## 4 Estimating sample size and power

### 4.1 Formulae for calculating power and sample size

Moerbeek and Wong 8 gave an expression for estimation of power for a partially nested design with a binary outcome measure based on the difference of proportions by using an inflation factor to account for partial nesting as follows:

(12) $$\left(1-\beta \right)=1-\Phi \left(z_{\left(1-\alpha /2\right)}-\frac{\pi_{G}-\pi_{C}}{SE\left[\pi_{G}-\pi_{C}\right]}\right)$$

with

(13) $$SE\left[\pi_{G}-\pi_{C}\right]=\sqrt{\frac{\pi_{C}\left(1-\pi_{C}\right)}{N_{C}}+\frac{\pi_{G}\left(1-\pi_{G}\right)}{N_{G}}\left(1+\left(m-1\right)\rho \right)}$$

where *m* is the cluster size and the sample size of subjects in each arm are *N*_*G*_ and *N*_*C*_. Assuming an allocation ratio between control and intervention of 1 : *λ*,

(14) $$N_{C}={\left(\frac{z_{\left(1-\alpha /2\right)}+z_{\left(1-\beta \right)}}{\pi_{G}-\pi_{C}}\right)}^{2}\left(\pi_{C}\left(1-\pi_{C}\right)+\frac{\pi_{G}\left(1-\pi_{G}\right)}{\lambda }\left(1+\left(m-1\right)\rho \right)\right)$$

from which the number of subjects in the group therapy arm and the therapy groups (*k*) in the group treatment arm can be determined by *N*_*G*_ = *λN*_*C*_ and *k* = *λN*_*C*_/*m*, respectively. We refer to this method as PROP in figures and tables.

When testing a new group‐based intervention, the investigators may wish to gain as much experience as possible with the new treatment. There may therefore be interest in randomising a greater proportion of subjects to the group treatment arm. In a standard trial design, unequal allocation may lead to loss of power, but this may not be the case here. Considering continuous outcomes, Roberts and Roberts (2005) suggested that greater power for a given total sample size of subjects can be achieved by using unequal allocation favouring the group arm. Minimising Equation (14) with respect to *λ* suggests $\lambda_{\max}=\sqrt{\frac{\pi_{G}\left(1-\pi_{G}\right)}{\pi_{C}\left(1-\pi_{C}\right)}\left(1+\left(m-1\right)\rho \right)}$ will give an optimal design in terms of total sample size. It can also be shown that $\lambda_{eq}=\frac{\pi_{G}\left(1-\pi_{G}\right)}{\pi_{C}\left(1-\pi_{C}\right)}\left(1+\left(m-1\right)\rho \right)$ gives the same power as equal allocation (*λ* = 1). Power will be increased relative to equal allocation where *λ* ∈ (1, *λ*_*eq*_).

Analyses using either the generalised estimating equation or the logistic random intercept models estimate log‐odds ratios. Suppose $\psi_{G}=\log_{e}\left[\frac{\pi_{G}}{\left(1-\pi_{G}\right)}\right]$ and $\psi_{C}=\log_{e}\left[\frac{\pi_{C}}{\left(1-\pi_{C}\right)}\right]$; one might estimate power by

(15) $$\left(1-\beta \right)=1-\Phi \left(z_{\left(1-\alpha /2\right)}-\frac{\psi_{G}-\psi_{C}}{SE\left[\psi_{G}-\psi_{C}\right]}\right)$$

with

(16) $$SE\left[\psi_{G}-\psi_{C}\right]=\sqrt{\frac{1}{N_{C}\pi_{C}\left(1-\pi_{C}\right)}+\frac{1+\left(m-1\right)\rho }{N_{G}\pi_{G}\left(1-\pi_{G}\right)}}\text{.}$$

With *N*_*G*_ = *λN*_*C*_, sample size in the control arm is therefore

(17) $$N_{C}={\left(\frac{z_{\left(1-\alpha /2\right)}+z_{\left(1-\beta \right)}}{\psi_{G}-\psi_{C}}\right)}^{2}\left(\frac{1}{\pi_{C}\left(1-\pi_{C}\right)}+\frac{\left(1+\left(m-1\right)\rho \right)}{\lambda \pi_{G}\left(1-\pi_{G}\right)}\right)$$

giving *N*_*G*_ = *λN*_*C*_ and *k* = *λN*_*C*_/*m*. We refer to this method as LOG‐ODDS. From (17), an optimal design for total sample size will be achieved when $\lambda_{\max}=\sqrt{\frac{\pi_{C}\left(1-\pi_{C}\right)}{\pi_{G}\left(1-\pi_{G}\right)}\left(1+\left(m-1\right)\rho \right)}$ for the LOG‐ODDS method and power will increase relative to equal allocation for *λ* ∈ (1, *λ*_*eq*_) where $\lambda_{eq}=\frac{\pi_{C}\left(1-\pi_{C}\right)}{\pi_{G}\left(1-\pi_{G}\right)}\left(1+\left(m-1\right)\rho \right)$.

Another approach, sometimes used to estimate sample size with binary data, is the arc‐sine transformation 38. Suppose $\gamma_{G}=2\arcsin\left[\sqrt{\pi_{G}}\right]$ and $\gamma_{C}=2\arcsin\left[\sqrt{\pi_{C}}\right]$; power would be estimated by

(18) $$\left(1-\beta \right)=1-\Phi \left(z_{\left(1-\alpha /2\right)}-\frac{\gamma_{G}-\gamma_{C}}{SE\left[\gamma_{G}-\gamma_{C}\right]}\right)$$

With

$$SE\left[\gamma_{G}-\gamma_{C}\right]=\sqrt{\frac{1}{N_{C}}+\frac{1+\left(m-1\right)\rho }{N_{G}}}\;\text{from}\;\text{which}$$

(19) $$N_{C}=\left(\frac{z_{\left(1-\alpha /2\right)}+z_{\left(1-\beta \right)}}{\gamma_{G}-\gamma_{C}}\right)\left(1+\frac{1+\left(m-1\right)\rho }{\lambda }\right)$$

with *N*_*G*_ = *λN*_*C*_ and *k* = *λN*_*C*_/*m*. We refer to this method as ARC‐SINE in tables. An optimal design in terms of total sample size is obtained for this method when $\lambda =\sqrt{1+\left(m-1\right)\rho }$, and power will increase relative to equal allocation for *λ* ∈ (1, *λ*_*eq*_) where *λ*_*eq*_ = 1 + (*m* − 1)*ρ*.

At this point, it is worth noting differences between Equations (14), (17) and (19). Equation (14) has terms involving the variance term *π*(1 − *π*) in the numerator, whereas Equation (17) has the same terms in the denominator, and the term is absent from Equation (19).

The formulae in the previous text for power and sample size all assume a normal approximation and so would be expected to overestimate power and underestimate sample size in small samples. Under methods of analysis, we considered the Satterthwaite test based on Equation (1) with degrees of freedom defined by Equation (6). The general form of the Satterthwaite test statistic is

$$T=\frac{\mu_{1}-\mu_{2}}{\sqrt{\frac{\sigma_{{}_{1}}^{2}}{n_{1}}+\frac{\sigma_{2}^{2}}{n_{2}}}},\text{with degrees of freedom}\;\upsilon =\frac{{\left(\frac{\sigma_{1}^{2}}{n_{1}}+\frac{\sigma_{2}^{2}}{n_{2}}\right)}^{2}}{\frac{{\left(\frac{\sigma_{1}^{2}}{n_{1}}\right)}^{2}}{n_{1}-1}+\frac{{\left(\frac{\sigma_{2}^{2}}{n_{2}}\right)}^{2}}{n_{2}-1}}\text{.}$$

To calculate power for the SATT method, we suggest setting $\mu_{1}=\pi_{G},\sigma_{1}^{2}=\frac{\pi_{G}\left(1-\pi_{G}\right)}{m}\left(1+\left(m-1\right)\rho \right),n_{1}=k,\mu_{2}=\pi_{C},\sigma_{2}^{2}=\pi_{C}\left(1-\pi_{C}\right)$, and *n*_2_ = *N*_*C*_ for a proportions‐based sample size calculation and $\mu_{1}=2\arcsin\left[\sqrt{\pi_{G}}\right],\sigma_{1}^{2}=\frac{\left(1+\left(m-1\right)\rho \right)}{m},n_{1}=k,\mu_{2}=2\arcsin\left[\sqrt{\pi_{C}}\right],\sigma_{2}^{2}=1$, and *n*_2_ = *N*_*C*_ for an arc‐sine‐based calculation. Moser *et al.* 39 give methods for estimating power for the Satterthwaite test that involve integration of the non‐central F‐distribution. These methods are referred to as PROP SATT and ARC‐SINE SATT in Tables S7–S12, respectively. All the methods of estimating sample size and power of equations described in the previous text including the Satterthwaite test method are implemented in a published user‐written stata routine called *clsampsi* 40.

### 4.2 Comparison of estimated and empirical power

To investigate the performance of the estimators of power and sample size described in the previous text, a third simulation study was carried out. Data generation and analysis methods were the same as in the study of small sample biases. The following parameter combinations were used: *ρ* = 0.05, 0.1; *m* = 5, 10; *N*_*G*_ = *N*_*C*_ = 100, 200, 400 and a selection of values of *π*_*G*_ ≠ *π*_*C*_. To restrict the size of the simulation study, pairs of *π*_*C*_and *π*_*G*_ were chosen that gave a power in the range [0.75, 0.95], based on Equation (12) or by the Satterthwaite test method with proportions, as combinations with power outside that range are of less interest when designing a trial. The calculated power for a combination {*π*_*G*_ = *a*, *π*_*C*_ = *b*} will be the same as the complimentary combination {*π*_*G*_ = 1 − *a*, *π*_*C*_ = 1 − *b*}. Because of partial nesting, the combination {*π*_*G*_ = *a*, *π*_*C*_ = *b*} does not give the same calculated power as the combination {*π*_*G*_ = *b*, *π*_*C*_ = *a*} unless *b* = 1 − *a*. We considered the following combinations, first, of *π*_*G*_ ≠ *π*_*C*_ ∈ {0.1, 0.15, 0.2,.., 0.5} and, secondly, of {*π*_*G*_ = 1 − *a*, *π*_*C*_ = 1 − *b*} and *π*_*C*_ ∈ {0.55, 0.6,.., 0.9}. This gives all unique scenarios out of all combinations of *π*_*G*_ ≠ *π*_*C*_ ∈ {0.1, 0.15,.., 0.9} of which 192 scenarios had a power in the required range. For each scenario, 40 000 simulations were carried out to give a 95% confidence interval of width less than ± 1 %across the specified range of power and ± 0.5 % where the power was 0.85%.

There were 478 simulation failures of the 7 680 000 simulations (0. 006%) of which 74% (353/478) occurred when the sample size in each arm was 100 and 67% (318/478) when the data‐generating rate in either arm was 0.1. Tables S7–S12 give the empirical power and calculated power for the methods being compared for all 192 scenarios. Considering empirical power, the discrepancy between the ATP, LGEE and LRI methods of analysis was greater with the larger cluster size (*m* = 10), larger values of the intra‐cluster correlation (ICC = 0.1) and values of *π*_*G*_ further from 0.5. Among the three methods of calculating power based on a normal approximation (PROP, ARC‐SINE and LOG‐ODDS) the greatest difference between methods was 14% between the PROP and LOG‐ODDS methods of calculation when *π*_*G*_ = 0.1, *π*_*C*_ = 0.3, *k* = 10, *m* = 10 and *ρ* = 0.1 (Table S8).

More important for sample size calculation is the relationship between empirical power and the calculated values as we would hope to identify methods of calculating power that give useful predictions of the empirical power for a particular analysis method. Table 2 gives the mean, minimum and maximum of the difference between empirical and calculated power. Negative values are of greatest concern as they indicate that the method of calculation is over‐optimistic compared with empirical power. The largest negative difference between the empirical power and a method of calculation was −14.1% between the likelihood ratio test (LRI (lrt)) and the proportions method (PROP), *π*_*G*_ = 0.1, *π*_*C*_ = 0.3, *k* = 10, *m* = 10 and *ρ* = 0.1 (Table S8). In contrast, the arc‐sine method of calculation gave excellent estimates of empirical power of the adjusted method of proportions with differences in the range (−1.5%, 0.5%), which suggests this to be a good method of calculation for this method of analysis.

**Table 2:** Difference between empirical power and calculated power.

Method of analysis 1	Method of calculation+ 2	Mean bias mean 1, 2 (%)	Overestimation min. 1, 2 (%)	Underestimation max. 1, 2 (%)
Adjusted test of proportions (ATP)	PROP	−0.7	−5.5	4.2
	ARC‐SINE	−0.2	−1.5	0.5
Satterthwaite *t*‐test (SATT)	SATT	−0.5	−3.3	3.2
	ARC‐SINE‐SATT	0.1	−3.9	3.0
Logistic generalised estimating equations (LGEE)	PROP	−0.5	−7.5	6.2
	ARC‐SINE	−0.0	−3.2	2.6
	LOG‐ODDS	1.6	−0.1	6.9
Logistic random intercept with Wald test (LRI (*z*))	PROP	−1.0	−7.8	4.6
	ARC‐SINE	−0.5	−3.5	0.9
	LOG‐ODDS	1.0	−0.5	6.6
Logistic random intercept (LRI) with likelihood ratio test (LRI (lrt))	PROP	−1.1	−14.1	5.9
	ARC‐SINE	−0.6	−9.9	1.8
	LOG‐ODDS	0.9	−0.3	3.6

+ See text for description of each method.

**Figure 6:**
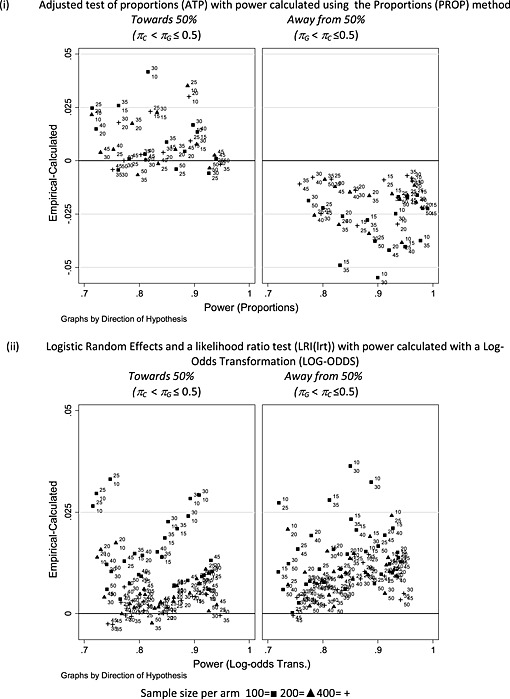
Comparison of empirical power of the adjusted test of proportions or logistic random intercept model.

2From inspection of Tables S7–S12, there is evidence that the direction of the difference between empirical and calculated power depends on the direction of the difference between *π_G_* and *π_C_*. The differences between the empirical power of the ATP and power calculated by the proportions method (PROP) according to the direct of the difference is displayed in Figure 6(i). Data‐generating values of *π_G_* and *π_C_* have been added to plotted values marked as superscript and subscripted, respectively. Note that with two cluster sizes (5 and 10) and two values of the intra‐cluster correlation coefficient, the figure may contain up to four points with the same *π_G_* and *π_C_* if several combinations had power in the range [75–95%]. Power calculated by the PROP method tends to underestimate empirical power for ATP when *π_C_* < *π_G_* and overestimate when *π_G_* < *π_C_* (Figure 6(i)). This effect will be reversed where *π_G_* and *π_C_* are greater than 0.5. A similar pattern is seen in corresponding figures (not given) when the PROP method is compared with the Satterthwaite test and logistic methods of analysis with the disparity being larger for the logistic methods.

Figure 6(ii) illustrates the empirical power of the logistic random intercept model with a likelihood ratio test and power calculation by the log‐odds transformation (LOG‐ODDS). In almost all instances, the log‐odds‐based calculation underestimates the empirical power. Similar effects are seen for the logistic generalised estimating equation model test (figure not given), although the underestimation of the log‐odds method is increased with a maximum of 6.9% for LGEE as compared with 3.6% for LRI with a likelihood ratio test. An explanation for the empirical power being greater than that by calculation using the LOG‐ODDS method could be the bias of the log‐odds ratio seen in Figure 4 for LGEE and Figure S2 for LRI.

In conclusion, we have seen that the arc‐sine method appears to be better than the proportions method where the analysis used is the adjusted test of proportions, and the log‐odds method of calculation gives a potentially useful lower bound for power where analysis is based on the logistic generalised estimation equations model or the logistic random intercept model. Differences in methods of calculation were greater in the circumstances that one would expected, namely larger cluster size and intra‐cluster correlation, smaller total sample size and the group treatment proportion *π_G_* being closer to either 0 or 1.

### 4.3 Implication of cluster size variation for power

To simplify the exposition, we assumed equal cluster sizes. Candel and Breukelen 41 investigated the effect of cluster size variation for a partially nested design with continuous outcome measures where the analysis is based on a linear mixed model. They conclude that there is a loss of efficiency, but this rarely exceeds 10% for what they considered to be a plausible variation in cluster size.

There is an extensive literature on the implications of cluster size variation in cluster randomised trials considering both continuous 34, 35, 36, 42 and binary 15, 30, 35 outcome measures. This shows that cluster size variation leads to loss of power, but the magnitude of this loss depends on how clusters are weighted in the analysis 34. Where analyses weight subjects equally, there is a simple adjustment to sample size formulae using the cluster size variance or the coefficient of variation (c.v.) of the cluster size. The sample size formulae given in Section 4.1 can be easily modified to take account of this type of weighting by adding terms for the coefficient of variation of clusters size 35, 42 or the cluster size variance to the design effect. This method of adjustment is implemented in the *clsampsi* routine 40 parameterised by the cluster size variance. Where the c.v. is small (<0.23), it has been shown that the loss of power is small 35. This is quite reassuring as the c.v. of cluster size will generally be small in trials of group‐administered treatments. For linear model‐based analyses of cluster randomised trials, Breukelen *et al.* 36 give a Taylor series approximation, again involving the coefficient of variation. They show that the effect of cluster size variation on efficiency rarely exceeds 10% for linear mixed models. Candel and Breukelen 30 consider the implications of varying cluster size for a cluster randomised trial with a binary outcome analysed using logistic random intercept models. They suggest that an inflation factor for sample size of 1.25 might be needed, but this is based on rather larger values of the coefficient of variation than are likely for group‐administered treatments. Whichever method of analysis is used, it would seem reasonable to assume that the effects of cluster size variation on power of trials of group‐administered treatment is not likely to be great, particularly as the cluster size variation is absent from the un‐clustered control arm of the trial.

## 5 Discussion

A strength of the simulation work presented here is that it was based on a design balanced in terms of total trial size, cluster size, intra‐cluster correlation and event rates, which simplifies interpretation of each of these factors separately. A limitation is that we have only considered the small cluster sizes one might expect in trials of group‐administered treatments and not the rather larger cluster sizes sometimes found in trials of therapist and care‐provider treatments. The implication of sample size can nevertheless be inferred by comparison of the effects for clusters of size 5 and 10. For example, the effect on test size was greater for a cluster size of 10 than 5 in all scenarios and so one can infer that performance will deteriorate further for larger cluster sizes.

A second limitation of the work is that equal cluster sizes have been assumed in the simulation studies. We do not consider this to be a major issue for trials of group‐administered treatments as treatment will generally specify a target therapy group size. In contrast, trials of therapist treatments may contain rather greater variation in cluster size, as the numbers of patients treated by each therapist may vary greatly due to differences in the numbers of therapists between clinics. What is more, the employment of trial therapists specifically to treat trial subjects may lead to a small number of particular large cluster sizes. Ideally, this should be avoided, but this variation may be quite difficult to control and so the coefficient of variation in therapist cluster size could be much larger than for group‐administered treatments. Not only would this have implications for power, but it might affect the performance of analysis methods. There is therefore the need for further work investigating the performance of methods in the presence of gross variation in cluster size, but this is not simply an issue for a partially nested trial but would also affect a fully nested trial comparing two or more therapist treatments.

We have compared several methods that might be applied to binary data from a partially nested trial design. Consideration of consistency for a null effect suggests a serious weakness of the logistic random coefficient model as defined by Equation (8) as a null effect on the scale of proportions corresponds to negative treatment effect on the scale of log‐odds where *π*_*G*_ = *π*_*C*_ < 0.5 and a positive effect for *π*_*G*_ = *π*_*C*_ > 0.5. The problem here is that the model compares a subject‐specific effect in the clustered arm with a marginal effect in the control.

In small samples, there was evidence of bias in the estimates of the treatment effects for logistic GEE and random intercept models. We also saw some test size bias for all methods. For a two‐sided test, the maximum test size for a 5% level test was only 7%, but this is deceptive as the type I error was not equally distributed between test sides with type I error raised for the alternative hypothesis that *π*_*G*_ < *π*_*C*_ where the null *π* < 0.5 and for *π*_*G*_ > *π*_*C*_ where the null *π*_*G*_ = *π*_*C*_ < 0.5. This bias was particularly striking for the summary measure test and the Satterthwaite test procedure. For one scenario, the type I error on one side was four times the type I error in the other side, while the type I error for the two‐sided test was only slightly elevated. This disparity between the test sizes can be explained by asymmetry in the data‐generating model and its consequences for the subsequent statistical analyses. As discussed in the previous text in Section 3.3, standard guidance in medical statistics is to carry out two‐sided tests 37 as this protects against analysis bias. While supporting this guidance, we have seen here an example where test performance may differ between sides due to asymmetry in the data‐generating model. Based on this experience, we would argue that it may be important to check the empirical test size in both tails separately where there is asymmetry in the data‐generating model or design.

The biases we have seen in empirical test size have in the main increased the probability of accepting of the alternate hypotheses *H*_1_ : *π*_*G*_ < *π*_*C*_ where *π*_*G*_ < *π*_*C*_ < 0.5 and *H*_1_ : *π*_*G*_ > *π*_*C*_ where *π*_*G*_ > *π*_*C*_ > 0.5. Based on the one‐sided test properties, the logistic models appeared to perform better than those based on proportions with the likelihood ratio test performing best in the range of scenarios considered here. This suggests that a likelihood ratio test may be the recommended procedure for this design within the range of design parameters we have considered. In extrapolating beyond this, one needs to be aware that a method of estimation may be affected by more than one source of bias that can interact. For example, the constraint that the random effects are non‐negative in a random effects model makes a test of the treatment effect more conservative. This will counter‐act the non‐conservative small sample bias giving better net performance for the method in some situations than others.

When the three methods of calculating power were compared with empirical power, we found both underestimation and overestimation. The arc‐sine method of calculating power was much closer to the empirical power of the adjusted test of proportions than that based on the proportions. The log‐odds method of calculation gave a lower bound for empirical power where the analysis was based on the logistic random intercept or logistic generalised estimating equations models. We therefore recommend the arc‐sine method for samples size calculation where the analysis is to be an adjusted test of proportions and the log‐odds methods where a logistic model is the planned analysis.

We also considered the implications of unequal allocation. Where the allocation ratio is in the range where *λ* ∈ (1, *λ*_*eq*_), power is increased compared with equal allocation. The range and optimal value of *λ*_max_ depend on the intra‐cluster correlation coefficient used in the sample size calculation. Should the estimated intra‐cluster correlation coefficient be smaller, power will be less for *π*_*G*_ < *π*_*C*_ < 0.5 than equal allocation. It will nevertheless be increased relative to the original calculation. If, instead, the intra‐cluster correlation is larger, the detrimental effect on power will be less than for equal allocation. Given that the implications of choosing too small an intra‐cluster correlation coefficient are of greater concern for sample size calculation than too large a value, an allocation ratio equal to*λ*_*eq*_ may have benefits over equal allocation, when designing partially nested trials.

## Supporting information



Supporting info item
