# Supplementary material for: Design and analysis of trials with a partially nested design and a binary outcome measure
Source: Stat Med. 2015 Dec 15;35(10):1616–36. doi: 10.1002/sim.6828 (PMC4949566; doi:10.1002/sim.6828)
Supplement: Supplementary file 1 — Supporting info item [file SIM-35-1616-s001.docx]

**Supporting Tables and Figures**

**Tables S1-S6** Empirical test size for two sided (nominal 5% level) and single sided (nominal 2.5% level) tests under the null (*π_C_=π_G_*) for cluster sizes of 5 and 10 and total sample size of 200, 400 and 800.

**Table S7-S12**  Empirical and calculated power for clusters of sizes 5 and 10 and total sample sizes of 200, 400 and 800.

**Figure S1** Bias in the treatment effect Log-Odds Ratio under the null (*π_C_=π_G_*) for the Logistic Random Intercept (LRI) model

**Figure S2** Bias in the treatment effect Log-Odds Ratio for the Logistic Random Intercept (LRI) model.

**Table S1** Empirical Test Size for two sided (nominal 5% level) and single sided (nominal 2.5% level) tests with 20 Clusters of 5 v 100 Controls. P is the proportion (*π_C_ = π_G_*).

|  | |  | Adjusted Test of  Proportions | | | Summary  Measure | | | Summary  Measure | | | Logistic  GEE | | | Logistic  Random Intercept | | | | | |
| --- | --- | --- | --- | --- | --- | --- | --- | --- | --- | --- | --- | --- | --- | --- | --- | --- | --- | --- | --- | --- |
|  | |  | (ATP) | | | (SMT) | | | (SATT) | | | (LGEE) | | | (LRI (z)) | | | (LRI (lrt)) | | |
| ICC% | P% | | Two% | Lower% | Upper% | Two% | Lower% | Upper% | Two% | Lower% | Upper% | Two% | Lower% | Upper% | Two% | Lower% | Upper% | Two% | Lower% | Upper% |
| 0 | | 10 | 5.2 | 2.6 | 2.7 | 5.4 | 2.6 | 2.8 | 4.8 | 2.4 | 2.4 | 4.5 | 2.3 | 2.2 | 3.6 | 1.7 | 1.9 | 4.4 | 2.0 | 2.5 |
|  | | 20 | 5.2 | 2.6 | 2.6 | 5.2 | 2.6 | 2.6 | 4.9 | 2.5 | 2.4 | 5.1 | 2.6 | 2.5 | 4.3 | 2.1 | 2.2 | 4.6 | 2.2 | 2.4 |
|  | | 30 | 5.7 | 2.9 | 2.8 | 5.8 | 2.9 | 2.9 | 5.4 | 2.8 | 2.6 | 5.6 | 2.9 | 2.7 | 4.7 | 2.3 | 2.4 | 4.9 | 2.4 | 2.5 |
|  | | 40 | 5.4 | 2.7 | 2.7 | 5.5 | 2.7 | 2.8 | 5.1 | 2.6 | 2.5 | 5.5 | 2.8 | 2.7 | 4.7 | 2.3 | 2.3 | 4.7 | 2.4 | 2.3 |
|  | | 50 | 5.3 | 2.7 | 2.6 | 5.5 | 2.8 | 2.7 | 5.0 | 2.6 | 2.5 | 5.4 | 2.7 | 2.7 | 4.8 | 2.4 | 2.4 | 4.9 | 2.5 | 2.4 |
|  | | 60 | 5.2 | 2.7 | 2.5 | 5.3 | 2.8 | 2.5 | 4.9 | 2.5 | 2.4 | 5.2 | 2.7 | 2.5 | 4.5 | 2.3 | 2.2 | 4.6 | 2.3 | 2.2 |
|  | | 70 | 5.1 | 2.6 | 2.5 | 5.2 | 2.6 | 2.6 | 4.8 | 2.4 | 2.4 | 5.0 | 2.4 | 2.6 | 4.1 | 2.1 | 2.0 | 4.3 | 2.3 | 2.1 |
|  | | 80 | 5.3 | 2.6 | 2.7 | 5.4 | 2.7 | 2.7 | 5.1 | 2.5 | 2.6 | 5.2 | 2.5 | 2.8 | 4.2 | 2.1 | 2.1 | 4.6 | 2.3 | 2.3 |
|  | | 90 | 5.1 | 2.6 | 2.5 | 5.3 | 2.8 | 2.5 | 4.7 | 2.4 | 2.4 | 4.5 | 2.1 | 2.3 | 3.5 | 1.8 | 1.7 | 4.3 | 2.3 | 2.0 |
| 5 | | 10 | 5.3 | 2.7 | 2.5 | 5.2 | 2.9 | 2.4 | 4.7 | 2.7 | 2.0 | 4.7 | 2.4 | 2.3 | 3.9 | 1.8 | 2.1 | 4.7 | 2.1 | 2.6 |
|  | | 20 | 5.4 | 2.9 | 2.5 | 5.4 | 3.0 | 2.5 | 5.0 | 2.8 | 2.2 | 5.3 | 2.8 | 2.5 | 4.6 | 2.3 | 2.3 | 5.0 | 2.5 | 2.5 |
|  | | 30 | 5.3 | 2.8 | 2.5 | 5.5 | 3.0 | 2.5 | 4.9 | 2.7 | 2.2 | 5.2 | 2.7 | 2.5 | 4.7 | 2.5 | 2.2 | 5.0 | 2.5 | 2.4 |
|  | | 40 | 5.2 | 2.6 | 2.6 | 5.3 | 2.7 | 2.6 | 4.9 | 2.5 | 2.4 | 5.2 | 2.6 | 2.6 | 4.8 | 2.4 | 2.4 | 4.9 | 2.4 | 2.5 |
|  | | 50 | 5.6 | 2.7 | 2.9 | 5.7 | 2.7 | 3.0 | 5.3 | 2.6 | 2.7 | 5.7 | 2.7 | 2.9 | 5.3 | 2.6 | 2.7 | 5.4 | 2.6 | 2.8 |
|  | | 60 | 5.5 | 2.9 | 2.6 | 5.6 | 3.0 | 2.7 | 5.3 | 2.7 | 2.6 | 5.6 | 2.9 | 2.7 | 5.1 | 2.7 | 2.4 | 5.2 | 2.8 | 2.4 |
|  | | 70 | 5.3 | 2.5 | 2.8 | 5.5 | 2.5 | 3.0 | 5.0 | 2.3 | 2.7 | 5.3 | 2.5 | 2.8 | 4.8 | 2.3 | 2.5 | 5.0 | 2.5 | 2.5 |
|  | | 80 | 5.2 | 2.5 | 2.7 | 5.3 | 2.5 | 2.8 | 4.9 | 2.2 | 2.7 | 5.2 | 2.5 | 2.7 | 4.6 | 2.3 | 2.3 | 5.0 | 2.6 | 2.4 |
|  | | 90 | 5.4 | 2.4 | 3.0 | 5.5 | 2.4 | 3.2 | 4.9 | 1.9 | 3.0 | 4.8 | 2.2 | 2.6 | 3.9 | 2.0 | 1.9 | 4.8 | 2.5 | 2.3 |
| 10 | | 10 | 5.7 | 3.3 | 2.5 | 5.6 | 3.5 | 2.1 | 5.0 | 3.3 | 1.6 | 5.3 | 2.8 | 2.4 | 4.3 | 2.3 | 2.0 | 5.2 | 2.5 | 2.7 |
|  | | 20 | 5.4 | 3.0 | 2.4 | 5.4 | 3.1 | 2.3 | 4.9 | 3.0 | 1.9 | 5.3 | 2.8 | 2.5 | 4.7 | 2.4 | 2.2 | 5.2 | 2.6 | 2.6 |
|  | | 30 | 5.4 | 2.8 | 2.6 | 5.5 | 3.0 | 2.5 | 5.0 | 2.7 | 2.3 | 5.4 | 2.7 | 2.7 | 4.9 | 2.5 | 2.5 | 5.3 | 2.6 | 2.7 |
|  | | 40 | 5.6 | 2.9 | 2.7 | 5.7 | 3.0 | 2.7 | 5.1 | 2.7 | 2.4 | 5.6 | 2.8 | 2.7 | 5.3 | 2.7 | 2.6 | 5.5 | 2.7 | 2.7 |
|  | | 50 | 5.6 | 3.0 | 2.7 | 5.7 | 3.0 | 2.7 | 5.3 | 2.8 | 2.5 | 5.6 | 3.0 | 2.7 | 5.4 | 2.8 | 2.6 | 5.5 | 2.9 | 2.6 |
|  | | 60 | 5.6 | 2.6 | 3.0 | 5.8 | 2.7 | 3.1 | 5.2 | 2.4 | 2.9 | 5.7 | 2.7 | 3.0 | 5.3 | 2.5 | 2.8 | 5.5 | 2.6 | 2.8 |
|  | | 70 | 5.5 | 2.5 | 3.0 | 5.7 | 2.4 | 3.2 | 5.1 | 2.2 | 3.0 | 5.4 | 2.6 | 2.9 | 4.9 | 2.3 | 2.6 | 5.2 | 2.6 | 2.7 |
|  | | 80 | 5.4 | 2.5 | 2.9 | 5.5 | 2.4 | 3.1 | 4.9 | 2.0 | 2.9 | 5.3 | 2.5 | 2.8 | 4.8 | 2.3 | 2.5 | 5.2 | 2.6 | 2.6 |
|  | | 90 | 5.8 | 2.3 | 3.5 | 5.8 | 2.1 | 3.7 | 5.1 | 1.6 | 3.5 | 5.3 | 2.3 | 3.0 | 4.4 | 2.0 | 2.4 | 5.3 | 2.7 | 2.7 |

**Table S2** Empirical Test Size for two sided (nominal 5% level) and single sided (nominal 2.5% level) tests with 10 Clusters of 10 v 100 Controls. P is the proportion (*π_C_ = π_G_*).

|  | |  | Adjusted Test of  Proportions | | | Summary  Measure | | | Summary  Measure | | | Logistic  GEE | | | Logistic  Random Intercept | | | | | |
| --- | --- | --- | --- | --- | --- | --- | --- | --- | --- | --- | --- | --- | --- | --- | --- | --- | --- | --- | --- | --- |
|  | |  | (ATP) | | | (SMT) | | | (SATT) | | | (LGEE) | | | (LRI (z)) | | | (LRI (lrt)) | | |
| ICC% | P% | | Two% | Lower% | Upper% | Two% | Lower% | Upper% | Two% | Lower% | Upper% | Two% | Lower% | Upper% | Two% | Lower% | Upper% | Two% | Lower% | Upper% |
| 0 | | 10 | 5.8 | 3.0 | 2.8 | 5.8 | 2.7 | 3.0 | 4.8 | 2.3 | 2.5 | 5.4 | 3.0 | 2.4 | 3.7 | 1.7 | 1.9 | 3.9 | 1.5 | 2.4 |
|  | | 20 | 5.4 | 2.8 | 2.6 | 5.5 | 2.7 | 2.8 | 4.7 | 2.5 | 2.3 | 5.6 | 3.1 | 2.5 | 4.1 | 2.1 | 2.0 | 4.1 | 2.0 | 2.1 |
|  | | 30 | 5.9 | 2.8 | 3.0 | 6.0 | 2.9 | 3.1 | 5.2 | 2.5 | 2.7 | 6.1 | 3.0 | 3.1 | 4.5 | 2.2 | 2.3 | 4.5 | 2.2 | 2.4 |
|  | | 40 | 5.6 | 2.7 | 2.9 | 5.6 | 2.7 | 3.0 | 5.0 | 2.4 | 2.6 | 5.8 | 2.9 | 3.0 | 4.4 | 2.1 | 2.4 | 4.3 | 2.0 | 2.3 |
|  | | 50 | 5.6 | 2.8 | 2.8 | 5.7 | 2.8 | 2.9 | 5.0 | 2.6 | 2.5 | 5.9 | 2.9 | 2.9 | 4.7 | 2.3 | 2.4 | 4.5 | 2.2 | 2.3 |
|  | | 60 | 5.9 | 3.0 | 2.9 | 5.9 | 3.1 | 2.9 | 5.3 | 2.7 | 2.6 | 6.2 | 3.1 | 3.0 | 4.7 | 2.4 | 2.3 | 4.6 | 2.4 | 2.2 |
|  | | 70 | 5.9 | 3.0 | 2.9 | 6.0 | 3.1 | 2.9 | 5.3 | 2.6 | 2.6 | 6.2 | 3.0 | 3.1 | 4.7 | 2.4 | 2.3 | 4.6 | 2.4 | 2.2 |
|  | | 80 | 5.6 | 2.9 | 2.7 | 5.8 | 3.1 | 2.6 | 5.0 | 2.6 | 2.3 | 5.7 | 2.8 | 2.9 | 4.1 | 2.2 | 1.9 | 4.2 | 2.3 | 1.9 |
|  | | 90 | 5.3 | 2.5 | 2.8 | 5.3 | 2.7 | 2.6 | 4.4 | 2.1 | 2.3 | 5.0 | 2.1 | 2.8 | 3.5 | 1.8 | 1.7 | 3.6 | 2.1 | 1.5 |
| 5 | | 10 | 6.2 | 3.8 | 2.4 | 6.1 | 3.8 | 2.2 | 4.9 | 3.3 | 1.5 | 6.0 | 3.5 | 2.5 | 4.7 | 2.5 | 2.2 | 4.6 | 1.9 | 2.7 |
|  | | 20 | 5.9 | 3.4 | 2.5 | 5.9 | 3.5 | 2.4 | 5.0 | 3.1 | 1.9 | 6.2 | 3.5 | 2.7 | 5.2 | 2.8 | 2.4 | 4.9 | 2.4 | 2.5 |
|  | | 30 | 6.0 | 3.2 | 2.7 | 6.0 | 3.3 | 2.7 | 5.0 | 2.9 | 2.1 | 6.3 | 3.4 | 3.0 | 5.4 | 2.8 | 2.6 | 5.2 | 2.6 | 2.6 |
|  | | 40 | 6.1 | 3.0 | 3.0 | 6.2 | 3.1 | 3.1 | 5.3 | 2.7 | 2.6 | 6.4 | 3.1 | 3.3 | 5.8 | 2.8 | 3.0 | 5.6 | 2.6 | 2.9 |
|  | | 50 | 6.0 | 3.0 | 3.0 | 6.1 | 3.1 | 3.1 | 5.2 | 2.7 | 2.6 | 6.4 | 3.2 | 3.2 | 5.9 | 3.0 | 2.9 | 5.5 | 2.8 | 2.8 |
|  | | 60 | 5.9 | 2.9 | 3.0 | 6.0 | 2.9 | 3.1 | 4.9 | 2.3 | 2.6 | 6.3 | 3.1 | 3.2 | 5.5 | 2.8 | 2.7 | 5.2 | 2.7 | 2.5 |
|  | | 70 | 6.1 | 2.7 | 3.4 | 6.1 | 2.7 | 3.4 | 5.0 | 2.1 | 3.0 | 6.4 | 2.9 | 3.5 | 5.5 | 2.6 | 2.9 | 5.2 | 2.5 | 2.6 |
|  | | 80 | 6.2 | 2.7 | 3.4 | 6.3 | 2.7 | 3.6 | 5.2 | 2.1 | 3.1 | 6.4 | 3.0 | 3.5 | 5.4 | 2.6 | 2.8 | 5.2 | 2.7 | 2.5 |
|  | | 90 | 6.2 | 2.3 | 3.8 | 6.2 | 2.2 | 4.0 | 5.0 | 1.6 | 3.4 | 6.0 | 2.4 | 3.6 | 4.8 | 2.2 | 2.6 | 4.5 | 2.6 | 1.9 |
| 10 | | 10 | 6.9 | 4.7 | 2.2 | 7.0 | 5.3 | 1.7 | 5.7 | 4.6 | 1.1 | 6.8 | 4.2 | 2.6 | 5.4 | 3.2 | 2.2 | 4.7 | 2.0 | 2.7 |
|  | | 20 | 6.4 | 4.1 | 2.4 | 6.4 | 4.3 | 2.1 | 5.4 | 3.9 | 1.5 | 6.7 | 4.0 | 2.8 | 5.9 | 3.5 | 2.4 | 5.6 | 3.0 | 2.6 |
|  | | 30 | 6.2 | 3.7 | 2.5 | 6.4 | 4.0 | 2.4 | 5.1 | 3.4 | 1.7 | 6.5 | 3.7 | 2.8 | 6.0 | 3.4 | 2.7 | 5.6 | 2.9 | 2.7 |
|  | | 40 | 6.0 | 3.2 | 2.8 | 6.2 | 3.4 | 2.8 | 5.1 | 2.8 | 2.3 | 6.4 | 3.4 | 3.0 | 5.9 | 3.1 | 2.9 | 5.6 | 2.8 | 2.7 |
|  | | 50 | 6.3 | 3.2 | 3.1 | 6.5 | 3.2 | 3.2 | 5.2 | 2.6 | 2.7 | 6.7 | 3.3 | 3.4 | 6.3 | 3.1 | 3.1 | 5.9 | 2.9 | 3.0 |
|  | | 60 | 6.5 | 3.0 | 3.4 | 6.7 | 3.1 | 3.6 | 5.4 | 2.4 | 3.0 | 6.8 | 3.3 | 3.6 | 6.4 | 3.1 | 3.3 | 6.0 | 3.1 | 3.0 |
|  | | 70 | 6.2 | 2.7 | 3.6 | 6.4 | 2.6 | 3.8 | 5.1 | 1.8 | 3.3 | 6.6 | 3.0 | 3.6 | 6.1 | 2.8 | 3.3 | 5.8 | 2.8 | 3.0 |
|  | | 80 | 6.5 | 2.4 | 4.0 | 6.5 | 2.2 | 4.3 | 5.5 | 1.6 | 3.9 | 6.9 | 2.9 | 4.0 | 6.1 | 2.6 | 3.5 | 5.8 | 2.8 | 3.0 |
|  | | 90 | 6.7 | 2.1 | 4.7 | 6.7 | 1.6 | 5.1 | 5.4 | 1.0 | 4.4 | 6.6 | 2.5 | 4.1 | 5.2 | 2.1 | 3.1 | 4.8 | 2.7 | 2.0 |

**Table S3** Empirical Test Size for two sided (nominal 5% level) and single sided (nominal 2.5% level) tests with 40 Clusters of 5 v 200 Controls. P is the proportion (*π_C_ = π_G_*).

|  | |  | Adjusted Test of  Proportions | | | Summary  Measure | | | Summary  Measure | | | Logistic  GEE | | | Logistic  Random Intercept | | | | | |
| --- | --- | --- | --- | --- | --- | --- | --- | --- | --- | --- | --- | --- | --- | --- | --- | --- | --- | --- | --- | --- |
|  | |  | (ATP) | | | (SMT) | | | (SATT) | | | (LGEE) | | | (LRI (z)) | | | (LRI (lrt)) | | |
| ICC% | P% | | Two% | Lower% | Upper% | Two% | Lower% | Upper% | Two% | Lower% | Upper% | Two% | Lower% | Upper% | Two% | Lower% | Upper% | Two% | Lower% | Upper% |
| 0 | | 10 | 5.2 | 2.5 | 2.7 | 5.3 | 2.5 | 2.8 | 5.0 | 2.4 | 2.7 | 5.0 | 2.4 | 2.6 | 4.5 | 2.1 | 2.4 | 5.0 | 2.3 | 2.7 |
|  | | 20 | 5.3 | 2.4 | 2.8 | 5.3 | 2.5 | 2.9 | 5.1 | 2.4 | 2.7 | 5.2 | 2.5 | 2.7 | 4.5 | 2.1 | 2.4 | 4.7 | 2.2 | 2.6 |
|  | | 30 | 5.1 | 2.6 | 2.5 | 5.2 | 2.6 | 2.6 | 5.0 | 2.5 | 2.4 | 5.1 | 2.6 | 2.5 | 4.5 | 2.3 | 2.2 | 4.7 | 2.4 | 2.3 |
|  | | 40 | 5.2 | 2.6 | 2.6 | 5.2 | 2.6 | 2.6 | 5.0 | 2.5 | 2.5 | 5.2 | 2.6 | 2.6 | 4.5 | 2.2 | 2.2 | 4.6 | 2.3 | 2.3 |
|  | | 50 | 5.3 | 2.7 | 2.6 | 5.4 | 2.7 | 2.6 | 5.1 | 2.7 | 2.5 | 5.3 | 2.7 | 2.6 | 4.8 | 2.5 | 2.3 | 4.9 | 2.5 | 2.4 |
|  | | 60 | 5.3 | 2.6 | 2.7 | 5.3 | 2.6 | 2.7 | 5.1 | 2.5 | 2.6 | 5.3 | 2.6 | 2.7 | 4.7 | 2.3 | 2.4 | 4.8 | 2.4 | 2.4 |
|  | | 70 | 5.3 | 2.7 | 2.7 | 5.3 | 2.7 | 2.7 | 5.1 | 2.6 | 2.6 | 5.3 | 2.6 | 2.7 | 4.7 | 2.3 | 2.4 | 4.8 | 2.4 | 2.4 |
|  | | 80 | 5.3 | 2.7 | 2.6 | 5.3 | 2.7 | 2.6 | 5.1 | 2.6 | 2.5 | 5.2 | 2.6 | 2.6 | 4.5 | 2.3 | 2.2 | 4.8 | 2.4 | 2.3 |
|  | | 90 | 4.8 | 2.4 | 2.4 | 4.9 | 2.5 | 2.4 | 4.7 | 2.4 | 2.3 | 4.6 | 2.3 | 2.3 | 4.1 | 2.1 | 2.0 | 4.5 | 2.4 | 2.2 |
| 5 | | 10 | 5.2 | 2.7 | 2.5 | 5.3 | 2.9 | 2.4 | 5.0 | 2.7 | 2.3 | 5.1 | 2.6 | 2.5 | 4.7 | 2.4 | 2.3 | 5.3 | 2.5 | 2.7 |
|  | | 20 | 5.2 | 2.6 | 2.6 | 5.2 | 2.7 | 2.5 | 5.0 | 2.6 | 2.4 | 5.2 | 2.6 | 2.6 | 4.9 | 2.4 | 2.5 | 5.1 | 2.5 | 2.6 |
|  | | 30 | 5.3 | 2.8 | 2.5 | 5.4 | 2.8 | 2.5 | 5.2 | 2.7 | 2.4 | 5.3 | 2.7 | 2.6 | 5.1 | 2.6 | 2.5 | 5.2 | 2.6 | 2.6 |
|  | | 40 | 5.1 | 2.5 | 2.6 | 5.2 | 2.6 | 2.6 | 4.9 | 2.5 | 2.4 | 5.1 | 2.5 | 2.6 | 4.8 | 2.3 | 2.5 | 4.9 | 2.4 | 2.5 |
|  | | 50 | 5.0 | 2.5 | 2.5 | 5.1 | 2.6 | 2.5 | 4.8 | 2.5 | 2.4 | 5.1 | 2.6 | 2.5 | 4.8 | 2.4 | 2.4 | 4.9 | 2.5 | 2.4 |
|  | | 60 | 5.3 | 2.5 | 2.7 | 5.3 | 2.5 | 2.8 | 5.1 | 2.4 | 2.7 | 5.3 | 2.6 | 2.8 | 5.0 | 2.4 | 2.6 | 5.1 | 2.5 | 2.6 |
|  | | 70 | 5.4 | 2.5 | 2.9 | 5.4 | 2.5 | 3.0 | 5.2 | 2.4 | 2.9 | 5.4 | 2.5 | 2.9 | 5.1 | 2.4 | 2.7 | 5.2 | 2.4 | 2.7 |
|  | | 80 | 5.2 | 2.5 | 2.7 | 5.3 | 2.5 | 2.7 | 5.1 | 2.4 | 2.7 | 5.2 | 2.6 | 2.7 | 4.9 | 2.4 | 2.5 | 5.1 | 2.6 | 2.5 |
|  | | 90 | 5.2 | 2.4 | 2.8 | 5.3 | 2.3 | 3.0 | 4.9 | 2.1 | 2.8 | 5.0 | 2.4 | 2.6 | 4.6 | 2.2 | 2.4 | 5.2 | 2.6 | 2.6 |
| 10 | | 10 | 5.1 | 2.9 | 2.2 | 5.0 | 3.1 | 1.9 | 4.7 | 2.9 | 1.8 | 5.0 | 2.7 | 2.3 | 4.7 | 2.6 | 2.0 | 5.1 | 2.7 | 2.4 |
|  | | 20 | 5.5 | 3.1 | 2.5 | 5.6 | 3.2 | 2.3 | 5.3 | 3.1 | 2.2 | 5.5 | 2.9 | 2.6 | 5.2 | 2.8 | 2.4 | 5.5 | 2.9 | 2.6 |
|  | | 30 | 5.2 | 2.7 | 2.5 | 5.3 | 2.8 | 2.5 | 5.1 | 2.7 | 2.4 | 5.2 | 2.6 | 2.6 | 5.0 | 2.5 | 2.5 | 5.1 | 2.6 | 2.6 |
|  | | 40 | 5.4 | 2.8 | 2.6 | 5.5 | 2.9 | 2.6 | 5.2 | 2.7 | 2.5 | 5.4 | 2.7 | 2.7 | 5.2 | 2.6 | 2.6 | 5.3 | 2.7 | 2.6 |
|  | | 50 | 5.2 | 2.5 | 2.7 | 5.3 | 2.5 | 2.7 | 5.1 | 2.5 | 2.6 | 5.2 | 2.5 | 2.7 | 5.0 | 2.5 | 2.6 | 5.1 | 2.5 | 2.6 |
|  | | 60 | 5.6 | 2.6 | 3.0 | 5.6 | 2.6 | 3.0 | 5.4 | 2.4 | 2.9 | 5.6 | 2.7 | 3.0 | 5.4 | 2.5 | 2.9 | 5.5 | 2.6 | 2.9 |
|  | | 70 | 5.3 | 2.5 | 2.8 | 5.3 | 2.4 | 2.9 | 5.1 | 2.3 | 2.8 | 5.3 | 2.6 | 2.7 | 5.1 | 2.4 | 2.6 | 5.2 | 2.5 | 2.7 |
|  | | 80 | 5.3 | 2.5 | 2.8 | 5.4 | 2.4 | 3.0 | 5.0 | 2.2 | 2.9 | 5.3 | 2.6 | 2.7 | 5.0 | 2.4 | 2.6 | 5.3 | 2.6 | 2.7 |
|  | | 90 | 5.3 | 2.3 | 3.0 | 5.3 | 2.1 | 3.2 | 5.0 | 1.9 | 3.1 | 5.2 | 2.4 | 2.8 | 4.9 | 2.2 | 2.7 | 5.3 | 2.6 | 2.7 |

**Table S4** Empirical Test Size for two sided (nominal 5% level) and single sided (nominal 2.5% level) tests with 20 Clusters of 10 v 200 Controls. P is the proportion (*π_C_ = π_G_*).

|  | |  | Adjusted Test of  Proportions | | | Summary  Measure | | | Summary  Measure | | | Logistic  GEE | | | Logistic  Random Intercept | | | | | |
| --- | --- | --- | --- | --- | --- | --- | --- | --- | --- | --- | --- | --- | --- | --- | --- | --- | --- | --- | --- | --- |
|  | |  | (ATP) | | | (SMT) | | | (SATT) | | | (LGEE) | | | (LRI (z)) | | | (LRI (lrt)) | | |
| ICC% | P% | | Two% | Lower% | Upper% | Two% | Lower% | Upper% | Two% | Lower% | Upper% | Two% | Lower% | Upper% | Two% | Lower% | Upper% | Two% | Lower% | Upper% |
| 0 | | 10 | 5.3 | 2.6 | 2.7 | 5.4 | 2.6 | 2.8 | 5.0 | 2.5 | 2.5 | 5.2 | 2.7 | 2.5 | 4.2 | 2.1 | 2.1 | 4.5 | 2.2 | 2.4 |
|  | | 20 | 5.5 | 2.9 | 2.6 | 5.6 | 2.9 | 2.7 | 5.2 | 2.7 | 2.5 | 5.6 | 3.0 | 2.6 | 4.6 | 2.4 | 2.2 | 4.7 | 2.4 | 2.3 |
|  | | 30 | 5.3 | 2.7 | 2.6 | 5.3 | 2.6 | 2.7 | 5.0 | 2.5 | 2.5 | 5.4 | 2.8 | 2.7 | 4.3 | 2.2 | 2.1 | 4.3 | 2.2 | 2.1 |
|  | | 40 | 5.5 | 2.8 | 2.7 | 5.6 | 2.8 | 2.8 | 5.2 | 2.7 | 2.5 | 5.7 | 2.9 | 2.8 | 4.6 | 2.4 | 2.2 | 4.6 | 2.4 | 2.2 |
|  | | 50 | 5.1 | 2.5 | 2.6 | 5.1 | 2.5 | 2.6 | 4.7 | 2.3 | 2.4 | 5.2 | 2.5 | 2.7 | 4.3 | 2.1 | 2.2 | 4.3 | 2.1 | 2.2 |
|  | | 60 | 5.3 | 2.6 | 2.7 | 5.4 | 2.6 | 2.8 | 5.0 | 2.4 | 2.6 | 5.5 | 2.7 | 2.8 | 4.4 | 2.1 | 2.3 | 4.4 | 2.1 | 2.2 |
|  | | 70 | 5.2 | 2.7 | 2.5 | 5.2 | 2.7 | 2.5 | 4.9 | 2.5 | 2.4 | 5.4 | 2.7 | 2.7 | 4.3 | 2.1 | 2.2 | 4.3 | 2.2 | 2.1 |
|  | | 80 | 5.5 | 2.7 | 2.8 | 5.5 | 2.7 | 2.8 | 5.1 | 2.5 | 2.6 | 5.5 | 2.7 | 2.8 | 4.5 | 2.3 | 2.2 | 4.6 | 2.4 | 2.2 |
|  | | 90 | 5.3 | 2.7 | 2.6 | 5.3 | 2.8 | 2.5 | 5.0 | 2.6 | 2.5 | 5.3 | 2.6 | 2.6 | 4.4 | 2.3 | 2.1 | 4.6 | 2.5 | 2.1 |
| 5 | | 10 | 5.7 | 3.4 | 2.3 | 5.6 | 3.6 | 2.0 | 5.2 | 3.5 | 1.7 | 5.8 | 3.3 | 2.5 | 5.2 | 2.9 | 2.3 | 5.3 | 2.7 | 2.6 |
|  | | 20 | 5.7 | 3.2 | 2.5 | 5.8 | 3.3 | 2.4 | 5.3 | 3.1 | 2.2 | 5.8 | 3.2 | 2.7 | 5.4 | 2.9 | 2.5 | 5.4 | 2.8 | 2.6 |
|  | | 30 | 5.5 | 2.8 | 2.6 | 5.5 | 3.0 | 2.6 | 4.9 | 2.7 | 2.3 | 5.6 | 2.9 | 2.8 | 5.4 | 2.7 | 2.7 | 5.2 | 2.5 | 2.7 |
|  | | 40 | 5.4 | 2.7 | 2.6 | 5.5 | 2.8 | 2.6 | 4.9 | 2.5 | 2.4 | 5.5 | 2.8 | 2.7 | 5.3 | 2.6 | 2.6 | 5.1 | 2.5 | 2.6 |
|  | | 50 | 5.6 | 2.8 | 2.8 | 5.7 | 2.9 | 2.8 | 5.2 | 2.6 | 2.6 | 5.8 | 2.9 | 2.9 | 5.6 | 2.8 | 2.7 | 5.4 | 2.8 | 2.7 |
|  | | 60 | 5.4 | 2.7 | 2.6 | 5.4 | 2.7 | 2.7 | 5.0 | 2.5 | 2.5 | 5.6 | 2.9 | 2.7 | 5.3 | 2.8 | 2.5 | 5.1 | 2.7 | 2.4 |
|  | | 70 | 5.2 | 2.5 | 2.7 | 5.2 | 2.4 | 2.8 | 4.8 | 2.2 | 2.6 | 5.4 | 2.6 | 2.7 | 5.1 | 2.5 | 2.6 | 5.0 | 2.5 | 2.5 |
|  | | 80 | 5.5 | 2.5 | 3.0 | 5.5 | 2.4 | 3.1 | 5.0 | 2.1 | 2.9 | 5.6 | 2.7 | 3.0 | 5.2 | 2.5 | 2.7 | 5.2 | 2.6 | 2.6 |
|  | | 90 | 5.5 | 2.2 | 3.3 | 5.4 | 1.9 | 3.5 | 5.1 | 1.7 | 3.4 | 5.5 | 2.4 | 3.2 | 5.0 | 2.2 | 2.9 | 5.0 | 2.4 | 2.6 |
| 10 | | 10 | 6.0 | 3.8 | 2.2 | 6.0 | 4.2 | 1.8 | 5.4 | 4.0 | 1.4 | 6.1 | 3.5 | 2.5 | 5.5 | 3.3 | 2.3 | 5.4 | 2.8 | 2.6 |
|  | | 20 | 5.5 | 3.3 | 2.2 | 5.5 | 3.6 | 1.9 | 5.0 | 3.4 | 1.6 | 5.7 | 3.2 | 2.5 | 5.4 | 3.1 | 2.3 | 5.3 | 2.8 | 2.5 |
|  | | 30 | 5.6 | 3.1 | 2.4 | 5.6 | 3.3 | 2.3 | 5.1 | 3.1 | 2.0 | 5.8 | 3.1 | 2.7 | 5.6 | 3.1 | 2.5 | 5.4 | 2.8 | 2.6 |
|  | | 40 | 5.3 | 2.7 | 2.6 | 5.4 | 2.8 | 2.5 | 4.9 | 2.6 | 2.3 | 5.5 | 2.8 | 2.7 | 5.4 | 2.7 | 2.7 | 5.3 | 2.6 | 2.7 |
|  | | 50 | 5.6 | 2.8 | 2.8 | 5.7 | 2.9 | 2.9 | 5.1 | 2.5 | 2.6 | 5.8 | 2.9 | 2.9 | 5.7 | 2.8 | 2.8 | 5.5 | 2.8 | 2.8 |
|  | | 60 | 5.4 | 2.6 | 2.8 | 5.5 | 2.6 | 3.0 | 5.0 | 2.3 | 2.7 | 5.6 | 2.7 | 2.9 | 5.5 | 2.7 | 2.8 | 5.3 | 2.6 | 2.7 |
|  | | 70 | 5.5 | 2.5 | 3.0 | 5.6 | 2.4 | 3.2 | 5.0 | 2.1 | 2.9 | 5.7 | 2.8 | 3.0 | 5.6 | 2.7 | 2.9 | 5.3 | 2.7 | 2.6 |
|  | | 80 | 5.6 | 2.3 | 3.3 | 5.6 | 2.1 | 3.5 | 5.1 | 1.8 | 3.3 | 5.8 | 2.6 | 3.1 | 5.5 | 2.4 | 3.1 | 5.4 | 2.6 | 2.8 |
|  | | 90 | 6.2 | 2.2 | 4.1 | 6.2 | 1.8 | 4.5 | 5.6 | 1.4 | 4.2 | 6.2 | 2.6 | 3.6 | 5.7 | 2.3 | 3.4 | 5.6 | 2.7 | 2.9 |

**Table S5** Empirical Test Size for two sided (nominal 5% level) and single sided (nominal 2.5% level) tests with 40 Clusters of 5 v 200 Controls. P is the proportion (*π_C_ = π_G_*).

|  | |  | Adjusted Test of  Proportions | | | Summary  Measure | | | Summary  Measure | | | Logistic  GEE | | | Logistic  Random Intercept | | | | | |
| --- | --- | --- | --- | --- | --- | --- | --- | --- | --- | --- | --- | --- | --- | --- | --- | --- | --- | --- | --- | --- |
|  | |  | (ATP) | | | (SMT) | | | (SATT) | | | (LGEE) | | | (LRI (z)) | | | (LRI (lrt)) | | |
| ICC% | P% | | Two% | Lower% | Upper% | Two% | Lower% | Upper% | Two% | Lower% | Upper% | Two% | Lower% | Upper% | Two% | Lower% | Upper% | Two% | Lower% | Upper% |
| 0 | | 10 | 5.3 | 2.8 | 2.5 | 5.3 | 2.8 | 2.5 | 5.2 | 2.8 | 2.5 | 5.2 | 2.7 | 2.4 | 4.7 | 2.5 | 2.2 | 4.9 | 2.5 | 2.3 |
|  | | 20 | 4.7 | 2.5 | 2.3 | 4.8 | 2.5 | 2.3 | 4.7 | 2.5 | 2.2 | 4.7 | 2.5 | 2.2 | 4.4 | 2.3 | 2.1 | 4.5 | 2.3 | 2.1 |
|  | | 30 | 5.1 | 2.5 | 2.6 | 5.1 | 2.5 | 2.6 | 5.0 | 2.5 | 2.5 | 5.1 | 2.5 | 2.6 | 4.7 | 2.3 | 2.4 | 4.7 | 2.3 | 2.4 |
|  | | 40 | 5.1 | 2.7 | 2.4 | 5.1 | 2.7 | 2.4 | 5.0 | 2.7 | 2.4 | 5.1 | 2.7 | 2.4 | 4.6 | 2.4 | 2.2 | 4.6 | 2.4 | 2.2 |
|  | | 50 | 5.0 | 2.5 | 2.5 | 5.0 | 2.5 | 2.5 | 4.9 | 2.5 | 2.5 | 5.0 | 2.5 | 2.5 | 4.7 | 2.4 | 2.3 | 4.7 | 2.4 | 2.4 |
|  | | 60 | 5.5 | 2.9 | 2.6 | 5.5 | 2.9 | 2.6 | 5.4 | 2.9 | 2.6 | 5.5 | 2.9 | 2.6 | 5.1 | 2.6 | 2.4 | 5.1 | 2.7 | 2.4 |
|  | | 70 | 4.9 | 2.3 | 2.6 | 4.9 | 2.3 | 2.6 | 4.7 | 2.3 | 2.5 | 4.9 | 2.3 | 2.6 | 4.4 | 2.1 | 2.3 | 4.5 | 2.1 | 2.4 |
|  | | 80 | 5.0 | 2.4 | 2.6 | 5.1 | 2.5 | 2.6 | 5.0 | 2.4 | 2.6 | 5.0 | 2.4 | 2.6 | 4.6 | 2.2 | 2.4 | 4.7 | 2.3 | 2.4 |
|  | | 90 | 5.0 | 2.5 | 2.6 | 5.1 | 2.5 | 2.6 | 5.0 | 2.4 | 2.6 | 4.9 | 2.4 | 2.5 | 4.5 | 2.2 | 2.3 | 4.7 | 2.3 | 2.4 |
| 5 | | 10 | 5.2 | 2.7 | 2.4 | 5.2 | 2.8 | 2.4 | 5.1 | 2.8 | 2.3 | 5.1 | 2.6 | 2.5 | 5.0 | 2.6 | 2.4 | 5.2 | 2.6 | 2.5 |
|  | | 20 | 4.9 | 2.6 | 2.3 | 5.0 | 2.7 | 2.3 | 4.9 | 2.6 | 2.2 | 4.9 | 2.5 | 2.4 | 4.8 | 2.4 | 2.3 | 4.9 | 2.5 | 2.4 |
|  | | 30 | 4.9 | 2.3 | 2.6 | 4.9 | 2.4 | 2.6 | 4.8 | 2.3 | 2.5 | 4.9 | 2.3 | 2.6 | 4.8 | 2.3 | 2.6 | 4.9 | 2.3 | 2.6 |
|  | | 40 | 5.0 | 2.5 | 2.6 | 5.1 | 2.5 | 2.6 | 4.9 | 2.4 | 2.5 | 5.1 | 2.5 | 2.6 | 4.9 | 2.4 | 2.5 | 5.0 | 2.4 | 2.6 |
|  | | 50 | 5.2 | 2.6 | 2.5 | 5.2 | 2.6 | 2.5 | 5.1 | 2.6 | 2.5 | 5.2 | 2.6 | 2.5 | 5.1 | 2.6 | 2.5 | 5.2 | 2.6 | 2.5 |
|  | | 60 | 5.1 | 2.5 | 2.7 | 5.2 | 2.5 | 2.7 | 5.0 | 2.4 | 2.6 | 5.1 | 2.5 | 2.7 | 5.0 | 2.4 | 2.6 | 5.1 | 2.4 | 2.6 |
|  | | 70 | 4.6 | 2.4 | 2.3 | 4.7 | 2.3 | 2.3 | 4.5 | 2.3 | 2.3 | 4.6 | 2.4 | 2.2 | 4.5 | 2.3 | 2.2 | 4.5 | 2.4 | 2.2 |
|  | | 80 | 5.0 | 2.4 | 2.7 | 5.1 | 2.3 | 2.7 | 4.9 | 2.3 | 2.7 | 5.0 | 2.4 | 2.6 | 4.9 | 2.3 | 2.5 | 5.0 | 2.4 | 2.6 |
|  | | 90 | 4.9 | 2.4 | 2.5 | 4.9 | 2.3 | 2.6 | 4.8 | 2.2 | 2.5 | 4.8 | 2.4 | 2.4 | 4.7 | 2.3 | 2.3 | 4.9 | 2.5 | 2.4 |
| 10 | | 10 | 5.2 | 2.9 | 2.3 | 5.3 | 3.1 | 2.2 | 5.1 | 3.0 | 2.1 | 5.1 | 2.6 | 2.5 | 4.9 | 2.6 | 2.3 | 5.2 | 2.6 | 2.6 |
|  | | 20 | 5.4 | 2.9 | 2.5 | 5.4 | 3.0 | 2.4 | 5.2 | 2.9 | 2.3 | 5.4 | 2.8 | 2.5 | 5.3 | 2.8 | 2.5 | 5.4 | 2.8 | 2.6 |
|  | | 30 | 4.8 | 2.4 | 2.4 | 4.8 | 2.5 | 2.3 | 4.7 | 2.5 | 2.2 | 4.7 | 2.3 | 2.4 | 4.7 | 2.3 | 2.3 | 4.7 | 2.3 | 2.4 |
|  | | 40 | 5.4 | 2.6 | 2.7 | 5.4 | 2.7 | 2.7 | 5.2 | 2.6 | 2.6 | 5.4 | 2.6 | 2.7 | 5.2 | 2.6 | 2.7 | 5.3 | 2.6 | 2.7 |
|  | | 50 | 4.9 | 2.4 | 2.5 | 5.0 | 2.5 | 2.5 | 4.9 | 2.4 | 2.5 | 4.9 | 2.4 | 2.5 | 4.9 | 2.4 | 2.5 | 5.0 | 2.5 | 2.5 |
|  | | 60 | 5.0 | 2.6 | 2.4 | 5.0 | 2.6 | 2.4 | 4.8 | 2.5 | 2.4 | 5.0 | 2.6 | 2.3 | 4.9 | 2.6 | 2.3 | 5.0 | 2.6 | 2.3 |
|  | | 70 | 4.9 | 2.3 | 2.6 | 4.9 | 2.2 | 2.7 | 4.8 | 2.2 | 2.6 | 4.9 | 2.4 | 2.6 | 4.8 | 2.3 | 2.5 | 4.9 | 2.4 | 2.5 |
|  | | 80 | 5.2 | 2.5 | 2.7 | 5.2 | 2.3 | 2.8 | 5.1 | 2.3 | 2.8 | 5.1 | 2.5 | 2.6 | 5.1 | 2.4 | 2.6 | 5.1 | 2.5 | 2.6 |
|  | | 90 | 5.2 | 2.3 | 2.9 | 5.1 | 2.1 | 3.0 | 5.0 | 2.0 | 3.0 | 5.1 | 2.4 | 2.7 | 5.0 | 2.3 | 2.7 | 5.2 | 2.5 | 2.7 |

**Table S6** Empirical Test Size for two sided (nominal 5% level) and single sided (nominal 2.5% level) tests with 40 Clusters of 10 v 400 Controls. P is the proportion (*π_C_ = π_G_*).

|  | |  | Adjusted Test of  Proportions | | | Summary  Measure | | | Summary  Measure | | | Logistic  GEE | | | Logistic  Random Intercept | | | | | |
| --- | --- | --- | --- | --- | --- | --- | --- | --- | --- | --- | --- | --- | --- | --- | --- | --- | --- | --- | --- | --- |
|  | |  | (ATP) | | | (SMT) | | | (SATT) | | | (LGEE) | | | (LRI (z)) | | | (LRI (lrt)) | | |
| ICC% | P% | | Two% | Lower% | Upper% | Two% | Lower% | Upper% | Two% | Lower% | Upper% | Two% | Lower% | Upper% | Two% | Lower% | Upper% | Two% | Lower% | Upper% |
| 0 | | 10 | 5.1 | 2.6 | 2.5 | 5.0 | 2.5 | 2.5 | 4.9 | 2.5 | 2.4 | 5.0 | 2.6 | 2.5 | 4.4 | 2.2 | 2.3 | 4.5 | 2.2 | 2.3 |
|  | | 20 | 5.4 | 2.8 | 2.7 | 5.5 | 2.7 | 2.7 | 5.2 | 2.7 | 2.6 | 5.5 | 2.8 | 2.7 | 4.8 | 2.4 | 2.4 | 4.8 | 2.4 | 2.4 |
|  | | 30 | 5.2 | 2.6 | 2.7 | 5.2 | 2.6 | 2.7 | 5.1 | 2.5 | 2.6 | 5.3 | 2.6 | 2.7 | 4.7 | 2.3 | 2.4 | 4.7 | 2.3 | 2.5 |
|  | | 40 | 5.2 | 2.7 | 2.5 | 5.2 | 2.7 | 2.5 | 5.0 | 2.6 | 2.4 | 5.3 | 2.7 | 2.6 | 4.5 | 2.3 | 2.2 | 4.5 | 2.3 | 2.2 |
|  | | 50 | 5.2 | 2.5 | 2.7 | 5.2 | 2.5 | 2.7 | 5.0 | 2.5 | 2.6 | 5.3 | 2.6 | 2.7 | 4.8 | 2.3 | 2.4 | 4.8 | 2.3 | 2.4 |
|  | | 60 | 5.1 | 2.5 | 2.6 | 5.1 | 2.5 | 2.6 | 4.9 | 2.4 | 2.5 | 5.1 | 2.5 | 2.6 | 4.5 | 2.2 | 2.2 | 4.4 | 2.2 | 2.2 |
|  | | 70 | 4.9 | 2.4 | 2.5 | 4.9 | 2.5 | 2.5 | 4.7 | 2.4 | 2.4 | 5.0 | 2.4 | 2.5 | 4.4 | 2.1 | 2.2 | 4.4 | 2.1 | 2.2 |
|  | | 80 | 5.1 | 2.5 | 2.7 | 5.2 | 2.5 | 2.7 | 4.9 | 2.4 | 2.6 | 5.2 | 2.5 | 2.8 | 4.6 | 2.2 | 2.4 | 4.7 | 2.3 | 2.4 |
|  | | 90 | 5.0 | 2.4 | 2.6 | 5.0 | 2.4 | 2.5 | 4.8 | 2.3 | 2.5 | 4.9 | 2.3 | 2.6 | 4.3 | 2.1 | 2.2 | 4.5 | 2.2 | 2.2 |
| 5 | | 10 | 5.2 | 3.0 | 2.2 | 5.2 | 3.1 | 2.1 | 4.9 | 3.0 | 1.9 | 5.2 | 2.9 | 2.4 | 5.1 | 2.8 | 2.3 | 5.0 | 2.6 | 2.4 |
|  | | 20 | 5.2 | 2.8 | 2.4 | 5.2 | 2.9 | 2.3 | 4.9 | 2.8 | 2.2 | 5.3 | 2.8 | 2.5 | 5.2 | 2.7 | 2.5 | 5.2 | 2.7 | 2.5 |
|  | | 30 | 5.0 | 2.6 | 2.4 | 5.1 | 2.7 | 2.4 | 4.8 | 2.5 | 2.3 | 5.1 | 2.6 | 2.5 | 5.1 | 2.6 | 2.5 | 5.0 | 2.5 | 2.5 |
|  | | 40 | 5.1 | 2.5 | 2.6 | 5.1 | 2.5 | 2.6 | 4.9 | 2.4 | 2.4 | 5.2 | 2.5 | 2.7 | 5.1 | 2.4 | 2.6 | 5.0 | 2.4 | 2.6 |
|  | | 50 | 5.2 | 2.5 | 2.8 | 5.3 | 2.5 | 2.8 | 5.0 | 2.4 | 2.6 | 5.3 | 2.5 | 2.8 | 5.3 | 2.5 | 2.8 | 5.2 | 2.5 | 2.7 |
|  | | 60 | 5.2 | 2.7 | 2.5 | 5.3 | 2.7 | 2.6 | 4.9 | 2.5 | 2.5 | 5.4 | 2.8 | 2.6 | 5.3 | 2.8 | 2.5 | 5.2 | 2.8 | 2.5 |
|  | | 70 | 5.3 | 2.5 | 2.8 | 5.3 | 2.5 | 2.8 | 5.0 | 2.3 | 2.7 | 5.4 | 2.7 | 2.7 | 5.3 | 2.6 | 2.7 | 5.2 | 2.6 | 2.6 |
|  | | 80 | 5.5 | 2.5 | 3.0 | 5.5 | 2.4 | 3.1 | 5.3 | 2.3 | 3.0 | 5.6 | 2.6 | 2.9 | 5.5 | 2.6 | 2.9 | 5.4 | 2.6 | 2.8 |
|  | | 90 | 5.4 | 2.3 | 3.1 | 5.3 | 2.1 | 3.2 | 5.1 | 1.9 | 3.2 | 5.4 | 2.5 | 2.9 | 5.2 | 2.4 | 2.9 | 5.2 | 2.6 | 2.7 |
| 10 | | 10 | 5.6 | 3.4 | 2.1 | 5.6 | 3.7 | 1.8 | 5.3 | 3.6 | 1.7 | 5.7 | 3.2 | 2.5 | 5.4 | 3.2 | 2.2 | 5.4 | 2.8 | 2.6 |
|  | | 20 | 5.3 | 2.9 | 2.4 | 5.4 | 3.2 | 2.2 | 5.1 | 3.0 | 2.0 | 5.4 | 2.8 | 2.6 | 5.3 | 2.8 | 2.5 | 5.3 | 2.7 | 2.7 |
|  | | 30 | 5.3 | 2.9 | 2.4 | 5.3 | 3.0 | 2.3 | 5.0 | 2.9 | 2.2 | 5.3 | 2.8 | 2.5 | 5.2 | 2.8 | 2.4 | 5.2 | 2.7 | 2.5 |
|  | | 40 | 5.3 | 2.7 | 2.6 | 5.4 | 2.8 | 2.6 | 5.0 | 2.6 | 2.4 | 5.3 | 2.7 | 2.6 | 5.3 | 2.7 | 2.6 | 5.2 | 2.6 | 2.6 |
|  | | 50 | 5.5 | 2.6 | 2.8 | 5.5 | 2.6 | 2.9 | 5.2 | 2.5 | 2.7 | 5.5 | 2.6 | 2.9 | 5.5 | 2.6 | 2.9 | 5.4 | 2.6 | 2.8 |
|  | | 60 | 5.3 | 2.3 | 3.0 | 5.4 | 2.3 | 3.1 | 5.1 | 2.2 | 2.9 | 5.4 | 2.4 | 3.0 | 5.4 | 2.4 | 3.0 | 5.3 | 2.4 | 2.9 |
|  | | 70 | 5.3 | 2.5 | 2.7 | 5.3 | 2.5 | 2.9 | 5.0 | 2.3 | 2.7 | 5.4 | 2.7 | 2.7 | 5.3 | 2.6 | 2.7 | 5.3 | 2.7 | 2.6 |
|  | | 80 | 5.1 | 2.2 | 2.9 | 5.1 | 2.0 | 3.1 | 4.8 | 1.8 | 3.0 | 5.3 | 2.5 | 2.8 | 5.1 | 2.3 | 2.8 | 5.1 | 2.5 | 2.6 |
|  | | 90 | 5.5 | 2.2 | 3.3 | 5.5 | 1.9 | 3.6 | 5.2 | 1.7 | 3.5 | 5.5 | 2.5 | 3.1 | 5.4 | 2.3 | 3.1 | 5.2 | 2.5 | 2.7 |

| **Table S7** Empirical and Calculated Power for 20 Clusters of 5 v 100 Controls. π_G_ and π_c_ are the proportions in the clustered and control arms respectively. | | | | | | | | | | | | | | | | | | | | | | | | | | | | | | | |  |  |
| --- | --- | --- | --- | --- | --- | --- | --- | --- | --- | --- | --- | --- | --- | --- | --- | --- | --- | --- | --- | --- | --- | --- | --- | --- | --- | --- | --- | --- | --- | --- | --- | --- | --- |
|  | | |  | | Empirical Power (z) | | | | | | | | | | | | | Calculated (z) | | | | | | | | Empir. | | | | Calculated | | | |
| $\pi_{G}\%$ | | | $\pi_{C}\%$ | | ATP | | | LGEE | | | LRI (z) | | | | LRI (lrt) | | | PROP | | | ARC  -SINE | | | LOG  -ODDS | | SATT | | | | PROP  SATT | | ARC-SINE  SATT | |
| **ICC=** | | **0.05** | | % | | % | | | % | | | | % | | | | % | | % | | | % | | | % | | | | % | | |  |  |
| 10 | | 25 | | 77.5 | | 75.9 | | | 74.3 | | | | 74.7 | | | | 78.8 | | 77.7 | | | 72.0 | | | 77.7 | | | | 77.9 | | | 76.2 |  |
| 10 | | 30 | | 93.3 | | 92.6 | | | 91.8 | | | | 92.0 | | | | 94.4 | | 93.5 | | | 88.8 | | | 93.4 | | | | 93.9 | | | 92.7 |  |
| 15 | | 35 | | 88.6 | | 87.8 | | | 87.2 | | | | 87.5 | | | | 90.0 | | 88.8 | | | 85.1 | | | 88.4 | | | | 89.3 | | | 87.6 |  |
| 20 | | 40 | | 84.4 | | 84.0 | | | 83.1 | | | | 83.5 | | | | 86.1 | | 84.6 | | | 81.9 | | | 84.1 | | | | 85.1 | | | 83.4 |  |
| 20 | | 45 | | 95.4 | | 95.3 | | | 94.9 | | | | 95.1 | | | | 96.5 | | 95.6 | | | 93.7 | | | 95.3 | | | | 96.1 | | | 94.9 |  |
| 25 | | 10 | | 77.6 | | 77.5 | | | 76.2 | | | | 78.0 | | | | 76.2 | | 77.7 | | | 74.7 | | | 75.2 | | | | 73.9 | | | 76.2 |  |
| 25 | | 45 | | 81.3 | | 81.1 | | | 80.1 | | | | 80.5 | | | | 82.9 | | 81.5 | | | 79.4 | | | 81.0 | | | | 81.8 | | | 80.1 |  |
| 25 | | 50 | | 93.9 | | 93.8 | | | 93.4 | | | | 93.6 | | | | 95.2 | | 94.2 | | | 92.6 | | | 93.7 | | | | 94.7 | | | 93.4 |  |
| 30 | | 10 | | 93.6 | | 93.6 | | | 93.0 | | | | 93.8 | | | | 92.8 | | 93.5 | | | 90.8 | | | 92.5 | | | | 91.3 | | | 92.7 |  |
| 30 | | 50 | | 79.1 | | 79.0 | | | 78.0 | | | | 78.4 | | | | 80.6 | | 79.2 | | | 77.6 | | | 78.6 | | | | 79.3 | | | 77.8 |  |
| 30 | | 55 | | 93.0 | | 93.0 | | | 92.7 | | | | 92.8 | | | | 94.3 | | 93.2 | | | 91.9 | | | 92.7 | | | | 93.6 | | | 92.3 |  |
| 35 | | 15 | | 88.7 | | 88.9 | | | 88.1 | | | | 89.0 | | | | 88.5 | | 88.8 | | | 86.9 | | | 87.4 | | | | 86.9 | | | 87.6 |  |
| 35 | | 55 | | 77.8 | | 77.8 | | | 76.9 | | | | 77.3 | | | | 79.2 | | 77.9 | | | 76.6 | | | 76.9 | | | | 77.8 | | | 76.5 |  |
| 35 | | 60 | | 92.6 | | 92.6 | | | 92.3 | | | | 92.5 | | | | 93.7 | | 92.7 | | | 91.5 | | | 92.1 | | | | 92.9 | | | 91.8 |  |
| 40 | | 20 | | 84.6 | | 84.8 | | | 83.9 | | | | 84.8 | | | | 84.8 | | 84.6 | | | 83.3 | | | 83.2 | | | | 83.1 | | | 83.4 |  |
| 40 | | 60 | | 77.8 | | 77.9 | | | 77.1 | | | | 77.5 | | | | 78.6 | | 77.5 | | | 76.4 | | | 76.7 | | | | 77.2 | | | 76.0 |  |
| 40 | | 65 | | 92.7 | | 92.8 | | | 92.4 | | | | 92.7 | | | | 93.6 | | 92.7 | | | 91.7 | | | 92.1 | | | | 92.7 | | | 91.8 |  |
| 45 | | 25 | | 81.4 | | 81.7 | | | 80.5 | | | | 81.3 | | | | 81.9 | | 81.5 | | | 80.3 | | | 80.1 | | | | 80.3 | | | 80.1 |  |
| 45 | | 65 | | 78.2 | | 78.3 | | | 77.3 | | | | 77.9 | | | | 78.9 | | 77.9 | | | 76.9 | | | 76.9 | | | | 77.3 | | | 76.5 |  |
| 45 | | 70 | | 93.0 | | 93.1 | | | 92.7 | | | | 92.9 | | | | 94.0 | | 93.2 | | | 92.2 | | | 92.3 | | | | 93.1 | | | 92.3 |  |
| 50 | | 25 | | 94.2 | | 94.4 | | | 93.8 | | | | 94.2 | | | | 94.7 | | 94.2 | | | 93.2 | | | 93.5 | | | | 93.9 | | | 93.4 |  |
| 50 | | 30 | | 79.2 | | 79.5 | | | 78.3 | | | | 79.0 | | | | 80.0 | | 79.2 | | | 78.2 | | | 77.9 | | | | 78.4 | | | 77.8 |  |
| **ICC=** | | **0.1** | |  | |  | | |  | | | |  | | | |  | |  | | |  | | |  |  | | | | | |  |  |
| 10 | | 30 | | 90.7 | | 89.3 | | | 88.7 | | | | 88.6 | | | | 93.2 | | 91.5 | | | 85.0 | | | 91.1 | | | 92.7 | | | | 90.4 |  |
| 15 | | 35 | | 85.3 | | 84.2 | | | 83.7 | | | | 84.0 | | | | 88.1 | | 86.0 | | | 81.2 | | | 85.4 | | | 87.2 | | | | 84.6 |  |
| 15 | | 40 | | 95.5 | | 95.0 | | | 94.9 | | | | 94.8 | | | | 97.2 | | 96.0 | | | 92.7 | | | 95.6 | | | 96.8 | | | | 95.3 |  |
| 20 | | 40 | | 80.9 | | 80.1 | | | 79.4 | | | | 79.8 | | | | 83.6 | | 81.4 | | | 77.9 | | | 80.8 | | | 82.4 | | | | 79.9 |  |
| 20 | | 45 | | 93.6 | | 93.3 | | | 92.9 | | | | 93.2 | | | | 95.4 | | 93.9 | | | 91.2 | | | 93.5 | | | 94.8 | | | | 93.0 |  |
| 25 | | 10 | | 73.9 | | 74.4 | | | 72.6 | | | | 75.2 | | | | 71.4 | | 74.1 | | | 72.2 | | | 70.3 | | | 68.9 | | | | 72.4 |  |
| 25 | | 45 | | 77.8 | | 77.3 | | | 76.5 | | | | 77.0 | | | | 80.0 | | 78.0 | | | 75.4 | | | 77.4 | | | 78.7 | | | | 76.4 |  |
| 25 | | 50 | | 92.0 | | 91.9 | | | 91.3 | | | | 91.7 | | | | 93.7 | | 92.2 | | | 90.0 | | | 91.8 | | | 93.0 | | | | 91.2 |  |
| 30 | | 10 | | 91.4 | | 91.7 | | | 90.9 | | | | 92.1 | | | | 89.8 | | 91.5 | | | 89.3 | | | 89.6 | | | 87.8 | | | | 90.4 |  |
| 30 | | 50 | | 75.5 | | 75.3 | | | 74.4 | | | | 74.9 | | | | 77.4 | | 75.7 | | | 73.7 | | | 74.9 | | | 75.9 | | | | 74.0 |  |
| 30 | | 55 | | 90.9 | | 90.8 | | | 90.3 | | | | 90.6 | | | | 92.5 | | 91.0 | | | 89.3 | | | 90.4 | | | 91.6 | | | | 89.9 |  |
| 35 | | 15 | | 85.8 | | 86.3 | | | 85.2 | | | | 86.5 | | | | 85.0 | | 86.0 | | | 84.7 | | | 83.8 | | | 83.0 | | | | 84.6 |  |
| 35 | | 55 | | 74.6 | | 74.6 | | | 73.6 | | | | 74.3 | | | | 75.8 | | 74.3 | | | 72.8 | | | 73.5 | | | 74.2 | | | | 72.7 |  |
| 35 | | 60 | | 90.2 | | 90.2 | | | 89.6 | | | | 90.1 | | | | 91.7 | | 90.4 | | | 89.0 | | | 89.5 | | | 90.7 | | | | 89.3 |  |
| 40 | | 20 | | 81.4 | | 81.8 | | | 80.7 | | | | 82.0 | | | | 81.1 | | 81.4 | | | 80.6 | | | 79.4 | | | 79.1 | | | | 79.9 |  |
| 40 | | 60 | | 74.1 | | 74.1 | | | 73.1 | | | | 73.9 | | | | 75.0 | | 73.9 | | | 72.7 | | | 72.7 | | | 73.4 | | | | 72.2 |  |
| 40 | | 65 | | 90.5 | | 90.6 | | | 90.0 | | | | 90.5 | | | | 91.5 | | 90.4 | | | 89.3 | | | 89.6 | | | 90.4 | | | | 89.3 |  |
| 45 | | 20 | | 94.1 | | 94.3 | | | 93.7 | | | | 94.3 | | | | 94.0 | | 93.9 | | | 93.0 | | | 93.1 | | | 92.8 | | | | 93.0 |  |
| 45 | | 25 | | 78.2 | | 78.6 | | | 77.4 | | | | 78.6 | | | | 78.1 | | 78.0 | | | 77.3 | | | 76.2 | | | 76.2 | | | | 76.4 |  |
| 45 | | 65 | | 74.8 | | 75.0 | | | 73.9 | | | | 74.9 | | | | 75.2 | | 74.3 | | | 73.4 | | | 73.1 | | | 73.4 | | | | 72.7 |  |
| 45 | | 70 | | 91.0 | | 91.2 | | | 90.6 | | | | 91.1 | | | | 91.8 | | 91.0 | | | 90.1 | | | 90.1 | | | 90.6 | | | | 89.9 |  |
| 50 | | 25 | | 92.1 | | 92.3 | | | 91.7 | | | | 92.3 | | | | 92.6 | | 92.2 | | | 91.3 | | | 91.1 | | | 91.5 | | | | 91.2 |  |
| 50 | | 30 | | 75.8 | | 76.1 | | | 75.0 | | | | 76.0 | | | | 76.2 | | 75.7 | | | 74.9 | | | 74.0 | | | 74.4 | | | | 90.4 |  |
| **Table S8** Empirical and Calculated Power for 10 Clusters of 10 v 100 Controls. π_G_ and π_c_ are the proportions in the clustered and control arms respectively. | | | | | | | | | | | | | | | | | | | | | | | | | | | | | | |  | | |
|  | | |  | Empirical Power (z) | | | | | | | | | | | | | Calculated (z) | | | | | | | | Empir. | | | Calculated | | | | | |
| $\pi_{G}\%$ | | | $\pi_{C}\%$ | ATP | | | | LGEE | | | | LRI (z) | | | | LRI (lrt) | PROP | | | | ARC  -SINE | | | LOG  -ODDS | SATT | | | PROP  SATT | | | | ARC-SINE  SATT | |
| **ICC=** | | **0.05** | | % | | % | | | % | | | | % | | | | % | | % | | | % | | | % | | | % | | | % | | |
| 10 | | 30 | | 90.1 | | 89.1 | | | 88.5 | | | | 84.6 | | | | 92.9 | | 90.9 | | | 84.0 | | | 90.1 | | | 91.8 | | | 88.4 | | |
| 15 | | 35 | | 84.7 | | 84.0 | | | 83.5 | | | | 81.0 | | | | 87.6 | | 85.3 | | | 80.2 | | | 84.0 | | | 85.8 | | | 82.1 | | |
| 15 | | 40 | | 94.9 | | 94.6 | | | 94.5 | | | | 93.3 | | | | 97.0 | | 95.6 | | | 92.1 | | | 94.6 | | | 96.2 | | | 93.9 | | |
| 20 | | 40 | | 80.5 | | 80.4 | | | 79.6 | | | | 77.7 | | | | 82.9 | | 80.6 | | | 76.9 | | | 79.3 | | | 80.6 | | | 77.3 | | |
| 20 | | 45 | | 92.9 | | 92.9 | | | 92.6 | | | | 91.6 | | | | 95.1 | | 93.5 | | | 90.6 | | | 92.2 | | | 93.8 | | | 91.3 | | |
| 25 | | 10 | | 73.1 | | 74.6 | | | 72.8 | | | | 74.2 | | | | 70.3 | | 73.2 | | | 71.6 | | | 65.9 | | | 64.8 | | | 69.6 | | |
| 25 | | 45 | | 76.8 | | 77.0 | | | 76.1 | | | | 74.5 | | | | 79.3 | | 77.2 | | | 74.4 | | | 75.0 | | | 76.5 | | | 73.7 | | |
| 25 | | 50 | | 91.2 | | 91.3 | | | 91.0 | | | | 90.1 | | | | 93.3 | | 91.7 | | | 89.4 | | | 90.0 | | | 91.6 | | | 89.3 | | |
| 30 | | 10 | | 90.6 | | 91.5 | | | 90.6 | | | | 91.3 | | | | 89.0 | | 90.9 | | | 88.9 | | | 86.2 | | | 84.3 | | | 88.4 | | |
| 30 | | 50 | | 74.9 | | 75.4 | | | 74.7 | | | | 73.4 | | | | 76.6 | | 74.8 | | | 72.8 | | | 72.7 | | | 73.5 | | | 71.3 | | |
| 30 | | 55 | | 90.1 | | 90.4 | | | 90.0 | | | | 89.3 | | | | 92.0 | | 90.5 | | | 88.6 | | | 88.4 | | | 89.9 | | | 87.9 | | |
| 35 | | 15 | | 85.1 | | 86.2 | | | 85.0 | | | | 85.5 | | | | 84.1 | | 85.3 | | | 84.1 | | | 80.4 | | | 79.4 | | | 82.1 | | |
| 35 | | 60 | | 89.6 | | 90.1 | | | 89.7 | | | | 89.2 | | | | 91.2 | | 89.9 | | | 88.4 | | | 87.8 | | | 88.8 | | | 87.2 | | |
| 40 | | 20 | | 80.5 | | 81.8 | | | 80.6 | | | | 80.8 | | | | 80.1 | | 80.6 | | | 79.9 | | | 75.8 | | | 75.7 | | | 77.3 | | |
| 40 | | 65 | | 89.4 | | 90.1 | | | 89.7 | | | | 89.3 | | | | 90.9 | | 89.9 | | | 88.7 | | | 87.4 | | | 88.3 | | | 87.2 | | |
| 45 | | 20 | | 93.0 | | 93.7 | | | 93.2 | | | | 93.2 | | | | 93.5 | | 93.5 | | | 92.6 | | | 90.5 | | | 90.5 | | | 91.3 | | |
| 45 | | 25 | | 76.8 | | 78.3 | | | 76.9 | | | | 76.9 | | | | 77.2 | | 77.2 | | | 76.6 | | | 72.5 | | | 73.0 | | | 73.7 | | |
| 45 | | 65 | | 73.3 | | 74.6 | | | 73.7 | | | | 73.1 | | | | 74.3 | | 73.5 | | | 72.6 | | | 69.8 | | | 70.5 | | | 69.9 | | |
| 45 | | 70 | | 89.9 | | 90.6 | | | 90.1 | | | | 89.9 | | | | 91.2 | | 90.5 | | | 89.5 | | | 87.7 | | | 88.4 | | | 87.9 | | |
| 50 | | 25 | | 91.5 | | 92.2 | | | 91.6 | | | | 91.6 | | | | 92.1 | | 91.7 | | | 90.9 | | | 89.1 | | | 89.2 | | | 89.3 | | |
| 50 | | 30 | | 74.8 | | 76.2 | | | 75.1 | | | | 74.7 | | | | 75.3 | | 74.8 | | | 74.1 | | | 70.9 | | | 71.3 | | | 71.3 | | |
| **ICC=** | | **0.1** | |  | | | | | | | | |  | | | |  | | | | | | | |  | | | | | |  | | |
| 10 | | 30 | | 84.5 | | 82.2 | | | 82.2 | | | | 75.9 | | | | 90.0 | | 85.7 | | | 75.6 | | | 85.0 | | | 88.3 | | | 82.0 | | |
| 10 | | 35 | | 94.0 | | 92.6 | | | 92.8 | | | | 89.3 | | | | 97.7 | | 95.5 | | | 87.8 | | | 94.2 | | | 97.1 | | | 93.4 | | |
| 15 | | 35 | | 78.2 | | 76.7 | | | 76.8 | | | | 72.9 | | | | 83.1 | | 78.9 | | | 71.9 | | | 77.7 | | | 80.5 | | | 74.8 | | |
| 15 | | 40 | | 90.9 | | 90.1 | | | 90.3 | | | | 88.1 | | | | 95.0 | | 92.1 | | | 86.1 | | | 90.6 | | | 93.6 | | | 89.2 | | |
| 15 | | 45 | | 96.8 | | 96.3 | | | 96.6 | | | | 95.4 | | | | 99.0 | | 97.8 | | | 94.1 | | | 96.5 | | | 98.5 | | | 96.3 | | |
| 20 | | 45 | | 87.8 | | 87.4 | | | 87.5 | | | | 85.9 | | | | 92.0 | | 89.1 | | | 84.4 | | | 86.9 | | | 89.9 | | | 85.7 | | |
| 20 | | 50 | | 95.8 | | 95.6 | | | 95.8 | | | | 95.1 | | | | 98.1 | | 96.6 | | | 93.6 | | | 95.2 | | | 97.2 | | | 94.7 | | |
| 25 | | 50 | | 85.7 | | 85.6 | | | 85.5 | | | | 84.3 | | | | 89.5 | | 86.8 | | | 83.2 | | | 84.0 | | | 86.8 | | | 83.1 | | |
| 25 | | 55 | | 94.7 | | 94.8 | | | 94.8 | | | | 94.3 | | | | 97.1 | | 95.6 | | | 93.2 | | | 93.7 | | | 95.8 | | | 93.5 | | |
| 30 | | 10 | | 85.8 | | 87.7 | | | 86.2 | | | | 87.5 | | | | 81.6 | | 85.7 | | | 85.2 | | | 78.1 | | | 75.4 | | | 82.0 | | |
| 30 | | 55 | | 84.5 | | 84.9 | | | 84.6 | | | | 83.6 | | | | 87.5 | | 85.2 | | | 82.6 | | | 82.2 | | | 84.3 | | | 81.4 | | |
| 30 | | 60 | | 94.2 | | 94.5 | | | 94.4 | | | | 94.0 | | | | 96.4 | | 94.9 | | | 93.1 | | | 92.9 | | | 94.6 | | | 92.7 | | |
| 35 | | 15 | | 78.8 | | 81.0 | | | 79.6 | | | | 80.6 | | | | 76.2 | | 78.9 | | | 79.2 | | | 71.3 | | | 70.4 | | | 74.8 | | |
| 35 | | 60 | | 84.0 | | 84.7 | | | 84.4 | | | | 83.8 | | | | 86.2 | | 84.4 | | | 82.5 | | | 81.2 | | | 82.7 | | | 80.6 | | |
| 35 | | 65 | | 94.1 | | 94.5 | | | 94.3 | | | | 94.1 | | | | 95.8 | | 94.7 | | | 93.4 | | | 92.4 | | | 93.8 | | | 92.4 | | |
| 40 | | 15 | | 91.9 | | 92.9 | | | 92.2 | | | | 92.7 | | | | 90.5 | | 92.1 | | | 91.7 | | | 87.6 | | | 86.1 | | | 89.2 | | |
| 40 | | 20 | | 73.7 | | 76.1 | | | 74.6 | | | | 75.3 | | | | 72.2 | | 73.8 | | | 74.1 | | | 66.8 | | | 66.8 | | | 69.5 | | |
| 40 | | 65 | | 83.9 | | 84.9 | | | 84.3 | | | | 84.0 | | | | 85.6 | | 84.4 | | | 83.1 | | | 80.6 | | | 81.8 | | | 80.6 | | |
| 40 | | 70 | | 94.4 | | 95.0 | | | 94.6 | | | | 94.6 | | | | 95.7 | | 94.9 | | | 94.0 | | | 92.4 | | | 93.4 | | | 92.7 | | |
| 45 | | 20 | | 88.7 | | 90.1 | | | 89.2 | | | | 89.6 | | | | 88.3 | | 89.1 | | | 88.8 | | | 84.2 | | | 83.8 | | | 85.7 | | |
| 45 | | 70 | | 84.7 | | 86.1 | | | 85.3 | | | | 85.2 | | | | 85.8 | | 85.2 | | | 84.4 | | | 80.9 | | | 81.7 | | | 81.4 | | |
| 50 | | 25 | | 86.3 | | 87.7 | | | 86.9 | | | | 87.0 | | | | 86.7 | | 86.8 | | | 86.3 | | | 82.0 | | | 82.4 | | | 83.1 | | |

| **Table S9** Empirical and Calculated Power for 40 Clusters of 5 v 200 Controls. π_G_ and π_c_ are the  proportions in the clustered and control arms respectively. | | | | | | | | | | | | | | | |  | | |  |
| --- | --- | --- | --- | --- | --- | --- | --- | --- | --- | --- | --- | --- | --- | --- | --- | --- | --- | --- | --- |
|  | |  | Empirical Power (z) | | | | | | | Calculated (z) | | | | | Empir. | Calculated | | | |
| $\pi_{G}\%$ | | $\pi_{C}\%$ | ATP | | LGEE | | LRI (z) | | LRI (lrt) | PROP | | ARC  -SINE | | LOG  -ODDS | SATT | PROP  SATT | ARC-SINE  SATT | | |
| **ICC=** | **0.05** | | % | % | | % | | % | | % | % | | % | | % | % | | % | |
| 10 | 20 | | 77.0 | 76.0 | | 75.4 | | 75.7 | | 78.0 | 77.2 | | 73.6 | | 77.3 | 77.5 | | 76.5 | |
| 15 | 30 | | 93.1 | 92.8 | | 92.7 | | 92.7 | | 94.1 | 93.4 | | 91.5 | | 93.1 | 93.8 | | 93.1 | |
| 20 | 10 | | 77.1 | 77.1 | | 76.5 | | 77.5 | | 75.9 | 77.2 | | 75.8 | | 75.6 | 74.9 | | 76.5 | |
| 20 | 35 | | 89.6 | 89.3 | | 89.1 | | 89.2 | | 90.6 | 89.8 | | 88.2 | | 89.6 | 90.2 | | 89.3 | |
| 25 | 40 | | 86.7 | 86.5 | | 86.1 | | 86.3 | | 87.5 | 86.7 | | 85.4 | | 86.6 | 87.0 | | 86.1 | |
| 30 | 15 | | 93.5 | 93.6 | | 93.3 | | 93.6 | | 93.1 | 93.4 | | 92.6 | | 93.0 | 92.6 | | 93.1 | |
| 30 | 45 | | 84.6 | 84.5 | | 84.1 | | 84.3 | | 85.1 | 84.3 | | 83.3 | | 84.4 | 84.6 | | 83.7 | |
| 35 | 20 | | 89.6 | 89.8 | | 89.4 | | 89.7 | | 89.7 | 89.8 | | 89.2 | | 89.1 | 89.1 | | 89.3 | |
| 35 | 50 | | 82.5 | 82.4 | | 82.0 | | 82.2 | | 83.5 | 82.8 | | 82.0 | | 82.2 | 82.9 | | 82.1 | |
| 40 | 25 | | 86.5 | 86.7 | | 86.2 | | 86.5 | | 86.8 | 86.7 | | 86.2 | | 85.8 | 86.1 | | 86.1 | |
| 40 | 55 | | 82.2 | 82.2 | | 81.8 | | 81.9 | | 82.6 | 82.0 | | 81.4 | | 81.8 | 82.0 | | 81.3 | |
| 45 | 30 | | 84.2 | 84.4 | | 83.9 | | 84.2 | | 84.6 | 84.3 | | 83.9 | | 83.7 | 83.9 | | 83.7 | |
| 45 | 60 | | 82.2 | 82.3 | | 81.8 | | 82.1 | | 82.5 | 82.0 | | 81.5 | | 81.8 | 81.9 | | 81.3 | |
| 50 | 35 | | 82.3 | 82.4 | | 81.8 | | 82.1 | | 83.2 | 82.8 | | 82.3 | | 81.7 | 82.5 | | 82.1 | |
| **ICC=** | **0.1** | |  | | | | |  | |  | | | | |  | | |  | |
| 10 | 25 | | 95.5 | 95.0 | | 95.1 | | 94.9 | | 96.6 | 95.8 | | 92.5 | | 95.7 | 96.5 | | 95.5 | |
| 15 | 30 | | 91.0 | 90.5 | | 90.5 | | 90.4 | | 92.6 | 91.3 | | 88.5 | | 91.2 | 92.2 | | 90.8 | |
| 20 | 10 | | 73.5 | 74.1 | | 73.1 | | 74.6 | | 71.4 | 73.6 | | 73.0 | | 71.2 | 70.2 | | 72.8 | |
| 20 | 35 | | 86.8 | 86.3 | | 86.3 | | 86.3 | | 88.4 | 87.1 | | 84.9 | | 86.9 | 88.0 | | 86.5 | |
| 25 | 40 | | 83.4 | 83.1 | | 82.9 | | 83.0 | | 84.9 | 83.7 | | 82.0 | | 83.4 | 84.3 | | 83.0 | |
| 30 | 15 | | 91.2 | 91.5 | | 91.1 | | 91.6 | | 90.4 | 91.3 | | 90.9 | | 90.5 | 89.7 | | 90.8 | |
| 30 | 45 | | 81.0 | 80.7 | | 80.4 | | 80.6 | | 82.2 | 81.1 | | 79.8 | | 80.8 | 81.6 | | 80.4 | |
| 35 | 20 | | 87.1 | 87.6 | | 86.9 | | 87.6 | | 86.6 | 87.1 | | 86.9 | | 86.2 | 85.8 | | 86.5 | |
| 35 | 50 | | 79.4 | 79.4 | | 79.0 | | 79.2 | | 80.3 | 79.4 | | 78.4 | | 79.1 | 79.6 | | 78.7 | |
| 40 | 25 | | 83.3 | 83.7 | | 83.1 | | 83.6 | | 83.4 | 83.7 | | 83.5 | | 82.4 | 82.6 | | 83.0 | |
| 40 | 55 | | 78.7 | 78.7 | | 78.3 | | 78.6 | | 79.3 | 78.6 | | 77.9 | | 78.2 | 78.6 | | 77.8 | |
| 45 | 30 | | 81.2 | 81.5 | | 80.9 | | 81.5 | | 81.2 | 81.1 | | 80.9 | | 80.4 | 80.3 | | 80.4 | |
| 45 | 60 | | 78.1 | 78.3 | | 77.8 | | 78.2 | | 79.1 | 78.6 | | 78.1 | | 77.6 | 78.3 | | 77.8 | |
| 50 | 35 | | 79.0 | 79.3 | | 78.8 | | 79.2 | | 79.7 | 79.4 | | 79.1 | | 78.3 | 78.9 | | 78.7 | |

| **Table S10** Empirical and Calculated Power for 20 Clusters of 10 v 200 Controls.π_G_ and π_c_ are the proportions in the clustered and control arms respectively. | | | | | | | | | | | | | | |  | | | | |  |  |
| --- | --- | --- | --- | --- | --- | --- | --- | --- | --- | --- | --- | --- | --- | --- | --- | --- | --- | --- | --- | --- | --- |
|  | |  | Empirical Power (z) | | | | | | | Calculated (z) | | | | | Empir. | | Calculated | | | | |
| $\pi_{G}\%$ | | $\pi_{C}\%$ | ATP | | LGEE | | LRI (z) | | LRI (lrt) | PROP | ARC  -SINE | | LOG  -ODDS | | SATT | | PROP  SATT | | ARC-SINE  SATT | | |
| **ICC=** | | **0.05** | % | % | | % | | % | | % | | % | | % | % | | | % | | % | |
| 10 | | 25 | 94.8 | 94.2 | | 94.4 | | 93.2 | | 96.4 | | 95.4 | | 91.9 | 95.0 | | | 96.1 | | 94.7 | |
| 15 | | 30 | 90.2 | 89.8 | | 89.8 | | 88.8 | | 92.2 | | 90.8 | | 87.8 | 90.2 | | | 91.5 | | 89.7 | |
| 20 | | 10 | 72.5 | 73.8 | | 72.8 | | 73.7 | | 70.3 | | 72.7 | | 72.4 | 69.0 | | | 67.9 | | 71.1 | |
| 20 | | 35 | 86.3 | 86.0 | | 86.0 | | 85.1 | | 87.9 | | 86.5 | | 84.1 | 86.1 | | | 86.9 | | 85.1 | |
| 25 | | 40 | 82.5 | 82.5 | | 82.3 | | 81.6 | | 84.3 | | 82.9 | | 81.1 | 82.1 | | | 83.1 | | 81.5 | |
| 30 | | 15 | 90.9 | 91.4 | | 91.1 | | 91.3 | | 89.8 | | 90.8 | | 90.4 | 89.3 | | | 88.1 | | 89.7 | |
| 30 | | 45 | 80.4 | 80.7 | | 80.4 | | 79.7 | | 81.5 | | 80.3 | | 78.9 | 79.7 | | | 80.1 | | 78.8 | |
| 35 | | 20 | 86.6 | 87.3 | | 86.9 | | 87.0 | | 85.8 | | 86.5 | | 86.3 | 84.8 | | | 84.1 | | 85.1 | |
| 35 | | 50 | 78.3 | 78.7 | | 78.4 | | 77.8 | | 79.6 | | 78.6 | | 77.6 | 77.4 | | | 78.1 | | 77.0 | |
| 40 | | 25 | 82.7 | 83.4 | | 83.0 | | 82.9 | | 82.6 | | 82.9 | | 82.8 | 80.9 | | | 80.9 | | 81.5 | |
| 40 | | 55 | 77.5 | 77.9 | | 77.6 | | 77.1 | | 78.5 | | 77.8 | | 77.0 | 76.3 | | | 77.0 | | 76.2 | |
| 45 | | 30 | 80.5 | 81.2 | | 80.7 | | 80.6 | | 80.3 | | 80.3 | | 80.1 | 78.8 | | | 78.6 | | 78.8 | |
| 45 | | 60 | 77.6 | 78.2 | | 77.8 | | 77.5 | | 78.3 | | 77.8 | | 77.3 | 76.3 | | | 76.7 | | 76.2 | |
| 50 | | 35 | 78.7 | 79.2 | | 78.8 | | 78.5 | | 78.9 | | 78.6 | | 78.3 | 77.1 | | | 77.2 | | 77.0 | |
| **ICC=** | | **0.1** |  | | | | |  | |  | | | | |  | | |  | |  | |
| 10 | | 25 | 90.5 | 89.0 | | 89.9 | | 87.1 | | 94.3 | | 91.8 | | 85.6 | 91.2 | | | 93.8 | | 90.6 | |
| 15 | | 30 | 85.0 | 83.7 | | 84.4 | | 82.3 | | 88.4 | | 85.6 | | 80.8 | 85.3 | | | 87.4 | | 83.9 | |
| 20 | | 35 | 79.9 | 79.1 | | 79.6 | | 77.7 | | 82.9 | | 80.3 | | 76.7 | 79.8 | | | 81.5 | | 78.5 | |
| 20 | | 40 | 95.0 | 94.7 | | 94.9 | | 94.3 | | 97.0 | | 95.7 | | 93.4 | 94.9 | | | 96.5 | | 94.8 | |
| 25 | | 10 | 92.3 | 93.2 | | 92.6 | | 93.2 | | 88.8 | | 91.8 | | 92.1 | 89.8 | | | 86.6 | | 90.6 | |
| 25 | | 40 | 75.9 | 75.5 | | 75.7 | | 74.3 | | 78.4 | | 76.3 | | 73.6 | 75.4 | | | 76.9 | | 74.4 | |
| 25 | | 45 | 93.7 | 93.6 | | 93.7 | | 93.2 | | 95.4 | | 94.0 | | 92.1 | 93.4 | | | 94.6 | | 93.0 | |
| 30 | | 15 | 85.5 | 86.8 | | 86.0 | | 86.7 | | 83.2 | | 85.6 | | 86.3 | 82.6 | | | 80.9 | | 83.9 | |
| 30 | | 50 | 92.1 | 92.2 | | 92.2 | | 91.8 | | 94.0 | | 92.8 | | 91.3 | 91.6 | | | 93.0 | | 91.6 | |
| 35 | | 20 | 80.5 | 81.7 | | 81.0 | | 81.5 | | 78.7 | | 80.3 | | 81.0 | 77.7 | | | 76.4 | | 78.5 | |
| 35 | | 55 | 91.7 | 91.8 | | 91.8 | | 91.5 | | 92.9 | | 92.0 | | 90.9 | 91.0 | | | 91.8 | | 90.7 | |
| 40 | | 25 | 75.8 | 77.2 | | 76.5 | | 76.8 | | 75.3 | | 76.3 | | 76.8 | 73.1 | | | 73.1 | | 74.4 | |
| 40 | | 60 | 91.2 | 91.5 | | 91.4 | | 91.2 | | 92.4 | | 91.7 | | 91.0 | 90.3 | | | 91.2 | | 90.4 | |
| 45 | | 25 | 93.9 | 94.4 | | 94.2 | | 94.3 | | 93.7 | | 94.0 | | 94.1 | 92.8 | | | 92.4 | | 93.0 | |
| 45 | | 30 | 73.4 | 74.5 | | 74.0 | | 74.1 | | 73.0 | | 73.4 | | 73.7 | 70.8 | | | 70.8 | | 71.5 | |
| 45 | | 65 | 91.7 | 92.1 | | 91.9 | | 91.8 | | 92.3 | | 92.0 | | 91.6 | 90.6 | | | 91.0 | | 90.7 | |
| 50 | | 30 | 92.4 | 92.9 | | 92.7 | | 92.7 | | 92.8 | | 92.8 | | 92.6 | 91.2 | | | 91.4 | | 91.6 | |

| **Table S11 Empirical** and Calculated Power for 80 Clusters of 5 v 400 Controls.π_G_ and π_c_ are the proportions in the clustered and control arms respectively. | | | | | | | | | | | | | | | | |  | | |  |  |
| --- | --- | --- | --- | --- | --- | --- | --- | --- | --- | --- | --- | --- | --- | --- | --- | --- | --- | --- | --- | --- | --- |
|  | |  | Empirical Power (z) | | | | | | | | Calculated (z) | | | | | Empir. | | Calculated | | | |
| $\pi_{G}\%$ | | $\pi_{C}\%$ | ATP | LGEE | | LRI (z) | | LRI (lrt) | | PROP | | ARC  -SINE | | LOG  -ODDS | | SATT | | PROP  SATT | ARC-SINE  SATT | | |
| **ICC=** | | **0.05** | % | | % | | % | | % | | % | | % | | % | % | | % | |  | |
| 15 | | 25 | 92.3 | | 92.1 | | 92.1 | | 92.1 | | 92.9 | | 92.4 | | 91.2 | 92.4 | | 92.7 | | 92.2 | |
| 20 | | 30 | 88.0 | | 87.7 | | 87.7 | | 87.7 | | 88.4 | | 87.9 | | 86.8 | 88.0 | | 88.2 | | 87.6 | |
| 25 | | 15 | 92.5 | | 92.5 | | 92.4 | | 92.6 | | 92.1 | | 92.4 | | 92.1 | 92.2 | | 91.8 | | 92.2 | |
| 25 | | 35 | 83.9 | | 83.7 | | 83.6 | | 83.6 | | 84.5 | | 83.9 | | 83.1 | 83.8 | | 84.2 | | 83.6 | |
| 30 | | 20 | 87.9 | | 88.0 | | 87.8 | | 88.0 | | 87.6 | | 87.9 | | 87.6 | 87.5 | | 87.3 | | 87.6 | |
| 30 | | 40 | 80.7 | | 80.5 | | 80.4 | | 80.5 | | 81.4 | | 80.9 | | 80.3 | 80.6 | | 81.1 | | 80.6 | |
| 35 | | 25 | 84.0 | | 84.1 | | 83.9 | | 84.0 | | 83.8 | | 83.9 | | 83.8 | 83.6 | | 83.5 | | 83.6 | |
| 35 | | 45 | 78.8 | | 78.8 | | 78.6 | | 78.6 | | 79.2 | | 78.8 | | 78.3 | 78.7 | | 78.9 | | 78.4 | |
| 40 | | 30 | 81.0 | | 81.2 | | 80.9 | | 81.0 | | 80.9 | | 80.9 | | 80.7 | 80.6 | | 80.5 | | 80.6 | |
| 40 | | 50 | 77.7 | | 77.6 | | 77.4 | | 77.5 | | 77.8 | | 77.5 | | 77.1 | 77.5 | | 77.5 | | 77.2 | |
| 45 | | 35 | 78.6 | | 78.6 | | 78.4 | | 78.5 | | 78.9 | | 78.8 | | 78.6 | 78.2 | | 78.5 | | 78.4 | |
| 45 | | 55 | 77.2 | | 77.3 | | 77.1 | | 77.2 | | 77.4 | | 77.1 | | 76.8 | 77.0 | | 77.0 | | 76.7 | |
| 50 | | 40 | 77.9 | | 78.0 | | 77.8 | | 77.9 | | 77.7 | | 77.5 | | 77.3 | 77.7 | | 77.3 | | 77.2 | |
| **ICC=** | | **0.1** |  | | | | | |  | |  | | | | |  |  | | |  | |
| 10 | | 20 | 95.3 | | 95.0 | | 95.1 | | 94.9 | | 96.2 | | 95.6 | | 93.5 | 95.5 | | 96.1 | | 95.4 | |
| 15 | | 25 | 90.2 | | 89.7 | | 89.9 | | 89.7 | | 91.1 | | 90.2 | | 88.3 | 90.5 | | 90.9 | | 89.9 | |
| 20 | | 30 | 84.5 | | 84.1 | | 84.1 | | 84.0 | | 85.9 | | 85.0 | | 83.5 | 84.7 | | 85.6 | | 84.6 | |
| 25 | | 15 | 90.2 | | 90.5 | | 90.2 | | 90.6 | | 89.3 | | 90.2 | | 90.2 | 89.7 | | 89.0 | | 89.9 | |
| 25 | | 35 | 80.7 | | 80.3 | | 80.3 | | 80.3 | | 81.5 | | 80.7 | | 79.5 | 80.7 | | 81.2 | | 80.3 | |
| 30 | | 20 | 84.7 | | 85.0 | | 84.7 | | 85.1 | | 84.3 | | 85.0 | | 85.1 | 84.1 | | 83.9 | | 84.6 | |
| 30 | | 40 | 77.4 | | 77.1 | | 77.0 | | 77.0 | | 78.1 | | 77.4 | | 76.6 | 77.4 | | 77.8 | | 77.1 | |
| 35 | | 25 | 80.6 | | 80.8 | | 80.5 | | 80.9 | | 80.3 | | 80.7 | | 80.8 | 79.9 | | 79.9 | | 80.3 | |
| 35 | | 45 | 74.7 | | 74.6 | | 74.5 | | 74.5 | | 75.8 | | 75.2 | | 74.6 | 74.6 | | 75.4 | | 74.8 | |
| 40 | | 30 | 77.2 | | 77.4 | | 77.2 | | 77.5 | | 77.2 | | 77.4 | | 77.5 | 76.7 | | 76.8 | | 77.1 | |
| 45 | | 35 | 74.8 | | 74.9 | | 74.7 | | 74.9 | | 75.2 | | 75.2 | | 75.1 | 74.3 | | 74.8 | | 74.8 | |

| **Table S12** Empirical and Calculated Power for 40 Clusters of 10 v 200 Controls. π_G_ and π_c_ are the proportions in the clustered and control arms respectively. | | | | | | | | | | |  | |  |  |
| --- | --- | --- | --- | --- | --- | --- | --- | --- | --- | --- | --- | --- | --- | --- |
|  | |  | Empirical Power (z) | | | | Calculated (z) | | | Empir. | Calculated | | | |
| $\pi_{G}\%$ | | $\pi_{C}\%$ | ATP | LGEE | LRI (z) | LRI (lrt) | PROP | ARC  -SINE | LOG  -ODDS | SATT | PROP  SATT | | ARC-SINE  SATT | |
| **ICC=** | | **0.05** | % | % | % | % | % | % | % | % | % | | % | |
| 10 | | 20 | 94.8 | 94.4 | 94.6 | 94.0 | 96.0 | 95.2 | 92.9 | 95.0 | 95.8 | | 94.9 | |
| 15 | | 25 | 89.5 | 89.1 | 89.2 | 88.6 | 90.6 | 89.6 | 87.5 | 89.6 | 90.2 | | 89.0 | |
| 20 | | 30 | 83.9 | 83.7 | 83.7 | 83.2 | 85.3 | 84.2 | 82.6 | 84.0 | 84.8 | | 83.6 | |
| 25 | | 15 | 89.7 | 90.1 | 89.9 | 90.1 | 88.6 | 89.6 | 89.7 | 88.9 | 87.8 | | 89.0 | |
| 25 | | 35 | 79.6 | 79.4 | 79.5 | 79.0 | 80.8 | 79.9 | 78.6 | 79.5 | 80.2 | | 79.1 | |
| 30 | | 20 | 84.0 | 84.5 | 84.3 | 84.4 | 83.5 | 84.2 | 84.4 | 83.0 | 82.7 | | 83.6 | |
| 30 | | 40 | 76.7 | 76.6 | 76.6 | 76.1 | 77.4 | 76.6 | 75.7 | 76.4 | 76.7 | | 75.8 | |
| 35 | | 25 | 79.8 | 80.4 | 80.1 | 80.2 | 79.4 | 79.9 | 80.0 | 78.9 | 78.6 | | 79.1 | |
| 40 | | 30 | 76.7 | 77.2 | 77.0 | 77.0 | 76.3 | 76.6 | 76.7 | 75.7 | 75.5 | | 75.8 | |
| 45 | | 35 | 73.9 | 74.4 | 74.2 | 74.1 | 74.3 | 74.3 | 74.3 | 73.1 | 73.5 | | 73.5 | |
| **ICC=** | | **0.1** |  | | |  |  | | |  |  | | | |
| 10 | | 20 | 90.6 | 89.5 | 90.2 | 88.6 | 93.5 | 91.5 | 87.3 | 91.2 | 93.2 | | 90.9 | |
| 15 | | 25 | 83.2 | 82.3 | 82.9 | 81.5 | 86.2 | 84.1 | 80.7 | 83.7 | 85.7 | | 83.3 | |
| 20 | | 10 | 92.0 | 92.7 | 92.3 | 92.7 | 89.0 | 91.5 | 92.3 | 90.6 | 88.1 | | 90.9 | |
| 20 | | 30 | 77.2 | 76.4 | 76.8 | 75.7 | 79.7 | 77.8 | 75.3 | 77.5 | 78.9 | | 76.9 | |
| 25 | | 15 | 84.3 | 85.2 | 84.7 | 85.2 | 82.0 | 84.1 | 85.1 | 82.5 | 81.0 | | 83.3 | |
| 30 | | 20 | 78.1 | 79.1 | 78.5 | 79.0 | 76.3 | 77.8 | 78.7 | 76.2 | 75.2 | | 76.9 | |
| 30 | | 45 | 95.4 | 95.3 | 95.4 | 95.2 | 96.2 | 95.5 | 94.7 | 95.3 | 95.9 | | 95.1 | |
| 35 | | 50 | 94.6 | 94.6 | 94.6 | 94.4 | 95.3 | 94.7 | 94.1 | 94.4 | 94.9 | | 94.3 | |
| 40 | | 55 | 94.3 | 94.3 | 94.3 | 94.2 | 94.7 | 94.3 | 93.9 | 94.0 | 94.3 | | 93.8 | |
| 45 | | 60 | 94.1 | 94.2 | 94.2 | 94.1 | 94.5 | 94.3 | 94.1 | 93.8 | 94.1 | | 93.8 | |
| 50 | | 35 | 94.6 | 94.7 | 94.6 | 94.6 | 94.7 | 94.7 | 94.7 | 94.2 | 94.3 | | 94.3 | |

| **Figure S1** Bias in the treatment effect Log-Odds Ratio estimate under the null (*π_C_=π_G_*) - Logistic Random Intercept (LRI) model as a function of proportion (*π_C_=π_G_*). | |
| --- | --- |
| 1. 20 Clusters of 5 + 100 Controls | 1. 10 Clusters of 10 + 100 Controls |
|  |  |
| 1. 40 Clusters of 5 + 200 Controls | 1. 20 Clusters of 10 + 200 Controls |
|  |  |
| 1. 80 Clusters of 5 + 400 Controls | 1. 40 Clusters of 10 + 400 Controls |
|  |  |
| 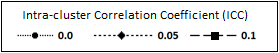 | |

| **Figure S2** Bias in the treatment effect Log-Odds Ratio estimate for the Logistic Random Intercept (LRI) model plotted against the data-generating value. Data-generating values of *π_G_* and *π_C_* are given where the bias is greater than 0.025. | |
| --- | --- |
| 1. 20 Clusters of 5 + 100 controls | 1. 10 Clusters of 10 + 100 controls |
|  |  |
| 1. 40 Clusters of 5 + 200 controls | 1. 20 Clusters of 10 + 200 controls |
|  |  |
| 1. 80 Clusters of 5 + 400 controls | 1. 40 Clusters of 10 + 400 controls |
|  |  |
